# Estrogen Receptor β-Selective Agonists Stimulate Calcium Oscillations in Human and Mouse Embryonic Stem Cell-Derived Neurons

**DOI:** 10.1371/journal.pone.0011791

**Published:** 2010-07-27

**Authors:** Lili Zhang, Brigitte E. Blackman, Marcus D. Schonemann, Tatjana Zogovic-Kapsalis, Xiaoyu Pan, Mary Tagliaferri, Heather A. Harris, Isaac Cohen, Renee A. Reijo Pera, Synthia H. Mellon, Richard I. Weiner, Dale C. Leitman

**Affiliations:** 1 Department of Obstetrics, Gynecology and Reproductive Sciences and Center for Reproductive Sciences, University of California San Francisco, San Francisco, California, United States of America; 2 Department of Molecular and Cellular Biology, University of California Davis, Davis, California, United States of America; 3 Bionovo Inc., Emeryville, California, United States of America; 4 Women's Health and Musculoskeletal Biology, Wyeth Research, Collegeville, Pennsylvania, United States of America; 5 Department of Obstetrics and Gynecology, Center for Human Embryonic Stem Cell Research and Education, Institute for Stem Cell Biology and Regenerative Medicine, Stanford University, Palo Alto, California, United States of America; 6 Department of Nutritional Science and Toxicology, University of California, Berkeley, California, United States of America; Vrije Universiteit Amsterdam, Netherlands

## Abstract

Estrogens are used extensively to treat hot flashes in menopausal women. Some of the beneficial effects of estrogens in hormone therapy on the brain might be due to nongenomic effects in neurons such as the rapid stimulation of calcium oscillations. Most studies have examined the nongenomic effects of estrogen receptors (ER) in primary neurons or brain slices from the rodent brain. However, these cells can not be maintained continuously in culture because neurons are post-mitotic. Neurons derived from embryonic stem cells could be a potential continuous, cell-based model to study nongenomic actions of estrogens in neurons if they are responsive to estrogens after differentiation. In this study ER-subtype specific estrogens were used to examine the role of ERα and ERβ on calcium oscillations in neurons derived from human (hES) and mouse embryonic stem cells. Unlike the undifferentiated hES cells the differentiated cells expressed neuronal markers, ERβ, but not ERα. The non-selective ER agonist 17β-estradiol (E_2_) rapidly increased [Ca2+]i oscillations and synchronizations within a few minutes. No change in calcium oscillations was observed with the selective ERα agonist 4,4′,4″-(4-Propyl-[1H]-pyrazole-1,3,5-triyl)trisphenol (PPT). In contrast, the selective ERβ agonists, 2,3-bis(4-Hydroxyphenyl)-propionitrile (DPN), MF101, and 2-(3-fluoro-4-hydroxyphenyl)-7-vinyl-1,3 benzoxazol-5-ol (ERB-041; WAY-202041) stimulated calcium oscillations similar to E_2_. The ERβ agonists also increased calcium oscillations and phosphorylated PKC, AKT and ERK1/2 in neurons derived from mouse ES cells, which was inhibited by nifedipine demonstrating that ERβ activates L-type voltage gated calcium channels to regulate neuronal activity. Our results demonstrate that ERβ signaling regulates nongenomic pathways in neurons derived from ES cells, and suggest that these cells might be useful to study the nongenomic mechanisms of estrogenic compounds.

## Introduction

Estrogens are critical for the development of reproductive organs and regulating reproductive function. They also are important in regulating the activity of non-reproductive tissues. The brain is one of the most important targets of estrogens. Estrogens have multiple effects in the brain, including neuronal development and maturation [1], synaptic plasticity [2] and excitability [3], neuroprotection and survival [4], and neurotransmitter and neuropeptide synthesis [5]. These actions of estrogens might lead to a modulation of cognition, memory, locomotor skills or mood [7], [8], [9], [10], [11]. In addition to the role in development and physiology, estrogens have been used therapeutically in the form of hormone therapy (HT) to treat hot flashes because of their effects on neurons involved in thermoregulation [6]. However, the Women's Health Initiative (WHI) trial found that the risks of HT exceeded its benefits [7]. The major problem associated with HT is that it increases the risk of cancer. The original HT regimen consisted of estrogen alone, but it was abandoned in women that had a hysterectomy because it increased the risk of endometrial cancer [14]. A progestin was then added to estrogens, which blocked the proliferative effects of estrogens on the endometrium. However, the WHI trial and multiple observational studies showed that progestins exacerbate the proliferative effects of estrogens on breast cells resulting in an increased risk of breast cancer [7], [8]. These findings have resulted in a marked decline in the use of HT [9], and created a large unmet need to discover safer estrogens for HT that lack the cancer-inducing properties, but retain the beneficial effects on menopausal symptoms. Estrogens used currently in HT are non-selective because they bind to and activate both the estrogen receptors, ERα [10] and ERβ [11]. This non-selective action leads to beneficial effects as well as the adverse effects. One potential way to improve HT is to discover estrogens that selectively regulate ERα or ERβ. Estrogens that selectively regulate ERβ appear to be a more attractive alternative to non-selective or ERα-selective estrogens for several reasons. First, numerous studies indicate that the proliferative effects of estrogens on breast and endometrial cells is mediated by ERα [12], [13]. ERα knockout mice (ERKO) exhibit only rudimentary development of the mammary gland and uterus because of the loss of the proliferative action of ERα [14]. Second, ERβ acts as a tumor suppressor that blocks ERα-mediated stimulation of breast cancer cells [15], [16]. ERβ agonists do not stimulate proliferation of human breast cancer cells, tumor formation in a mouse xenograft model or mammary epithelial cell proliferation in rats [17], [18], [19]. Furthermore, ERβ agonists do not increase uterine size in rodents [17], [18], [19]. Third, while the role of ERβ in hot flash prevention is unclear, the ERβ agonist, 2,3-bis(4-Hydroxyphenyl)-propionitrile (DPN) reduced hot flashes in a rat model [20]. However, other ERβ agonists were ineffective in a different rat hot flash model [21]. Using ERα and ERβ (BERKO) knockout mice it was found that both ERs are involved hot flash prevention using the tail skin temperature as a surrogate marker for hot flashes [22]. A clinical trial with the plant-derived ERβ-selective extract, MF101 found a significant reduction of hot flashes in postmenopausal women [23]. Taken together, these results suggest that ERβ agonists will not promote breast or uterine cancer, but they might retain the beneficial effects of estrogens on hot flash prevention.

The future development of ERβ agonists for HT to treat menopausal symptoms requires a greater understanding of the physiological roles of ERβ and mechanism of action of ERβ agonists in neurons. One obstacle to investigating the actions and roles of ERβ in neurons is the lack of cell based models. Most studies have examined the effects of estrogens on primary neurons or brain slices derived from animals. Although primary neurons cannot be grown continuously in culture, these important studies have shown that ERs are involved in nongenomic regulation of neuronal activity. It was first demonstrated that estrogens altered the firing rate of neurons after a few minutes [24]. Other studies have confirmed the rapid effects of estrogens on neurons [25], [26], [27] and other cell types [28]. With the introduction of ERα-selective agonists, such as 4,4′,4″-(4-Propyl-[1H]-pyrazole-1,3,5-triyl)trisphenol (PPT) [29] and ERβ-selective agonists, such as DPN [30], ERB-041 [31], and natural ERβ agonists derived from plants [19] it has become possible to investigate the role of ERα and ERβ in mediating the nongenomic effects of estrogens in neurons. In rat hippocampal neurons both PPT and DPN induced a rapid increase in intracellular Ca2+ influx [32], suggesting that both ERs regulate calcium influx. In GnRH neurons, estradiol (E_2_) and DPN rapidly increased the firing rate, whereas PPT did not have any effect on neuronal activity [33]. These studies indicate that ERβ has an important role in mediating nongenomic actions in neurons, which is consistent with the observations that ERβ is present in the hippocampus and hypothalamus [34], [35]. Besides using primary neurons or brain slices from animals, a neuronal cell line that can be continuously maintained in culture is the GT-1 cell line, which is an immortalized gonadotropin-releasing hormone (GnRH)-secreting cell line derived from transgenic mice [36]. This cell line has been used to study the neuronal effects of estrogens [37], but the physiological relevance of an immortalized cell line can always be questioned. Furthermore, whether the effects on GnRH neurons reflect actions of estrogens on other neurotransmitter containing neurons is unknown. An alterative and potentially important model to study the effects of estrogens is to use neurons derived from embryonic stem (ES) cells. ES cells are pluripotent cells derived from the inner cell mass of blastocysts that can differentiate into any cell type [38], [39]. A major advantage of ES cells is that they can be used to generate a continuous supply of neurons [40]. The goal of these studies was to use neurons derived from ES cells as an alternative model to study the nongenomic action of ERβ on neurons. We examined the role of ERβ on calcium oscillations by comparing the non-selective ER agonist, E_2_, with the ERβ-selective agonists, DPN [30], ERB-041 [17], [31], and MF101 [19]. Using these selective ER subtype agonists, we show that ERβ mediates the stimulation of calcium oscillations in neurons derived from mouse and human ES cells, which demonstrates that these neurons might be a useful cell based model to study the role of ERs and the nongenomic mechanisms of estrogens.

## Materials and Methods

### Reagents

Veratridine (VTD), tetrodotoxin (TTX), 6-(O-carboxymethyl)oxime (E_2_-BSA), nifedipine (Nif), ω-agatoxin IVA (AgTx), 17-β estradiol (E_2_), and ω-conotoxin GVIA (CgTx) were purchased from Sigma-Aldrich. DPN and PPT were purchased from Tocris Bioscience. U0126 and Ly294,002 were purchased from Cell Signaling Technology. MF101 was obtained from Bionovo Inc. 2-(3-fluoro-4-hydroxyphenyl)-7-vinyl-1,3-benzoxazol-5-ol (ERB-041) was from the Wyeth Research compound library.

### Human embryonic stem cell culture and neuronal differentiation

All experiments involving human embryonic stem (hES) cells were approved by the University of California, San Francisco Embryonic Stem Cell Research Oversight Committee and the Institutional Review Board of California. NIH registered human ES (hES) cell lines, H9 and H7 were obtained from WiCell. Human ES cells were maintained and differentiated into neurons using a protocol modified from a previous report [41]. In brief, hES cells were expanded on irradiated mouse embryonic fibroblasts in a knockout serum replacer (KSR) medium, which is DMEM with high glucose supplemented with 20% KSR, 10 ng/ml fibroblast growth factor (FGF, R&D Systems), 2 mM glutamine, 0.1 mM non-essential amino acids (NEAA), 100 units/ml penicillin and streptomycin and 55 µM β-mercaptoethanol. The medium was changed daily and confluent hES cells were passaged every 4–6 days by incubation with 1 mg/ml collagenase IV for 10 min at 37°C. To convert hES cells into human neuronal progenitor cells (hNPCs) by embryonic body (EB) formation, the cells were dissociated into cell aggregates with collagenase IV, replated into low attachment 6-well plates and cultured in hNPC medium, which is a 1∶1 mixture of the KSR medium and neurobasal medium supplemented with N2 supplements, 10 µM retinoic acid (RA, Sigma-Aldrich), 500 ng/ml epidermal growth factor (EGF, R&D systems), 2 mM glutamine, 0.1 mM NEAA and 100 units/ml penicillin and streptomycin. After 10 days in suspension culture with the medium changed every other day the EBs were dissociated into single cells using the Neural Tissue Dissociation (papain) Kit (Myltenyi Biotec), plated into Matrigel coated 6-well dishes at 40–50% confluency and were expanded in the hNPC medium without RA for 2–3 passages. To convert human neural progenitor cells (hNPCs) into human neurons, hNPCs were passaged by papain and replated in Matrigel coated 6-well dishes at 60–70% confluency. Human neurons were obtained after 2–3 weeks of culture in human differentiation neurobasal medium (hDFNB), which is neurobasal medium supplemented with N2 supplements, 2 mM glutamine, 0.1 mM NEAA and 100 units/ml penicillin and streptomycin. All experiments were done during the third week of culturing in the hDFNB. All materials were purchased from Invitrogen unless specified.

### Mouse embryonic stem cell culture and neuronal differentiation

The mouse embryonic stem (mES) cell line ES14 was obtained from Dr. Nigel Killeen at the University of California, San Francisco. The cells were grown in Glasgow Minimum Essential Medium (Sigma-Aldrich) supplemented with 15% fetal bovine serum (FBS, Hyclone), 1000 u/ml leukemia inhibitory factor (Millipore), 2 mM glutamine, 0.1 mM NEAA, 1 mM sodium pyruvate, 100 units/ml penicillin and streptomycin and 55 µM β-mercaptoethanol with daily medium change. Cells were passaged onto 0.1% gelatin-coated dishes and expanded in feeder free condition. The production of neurons was done using a variation of the Bain method [53]. To induce EB formation, the cells were trypsinized into cell aggregates and plated at 4 million/10 cm bacterial petri dish in DFNB medium. EBs were suspended in DFNB containing 1 µM RA for 4 days and the medium was changed every other day. The 4-day-old EBs were trypsinized into a single-cell suspension and replated at 80–90% confluency on dishes or 25 mm glass coverslips coated with 15 µg/ml polyornithine (Sigma-Aldrich) and 1 µg/ml fibronectin (R&D Systems). Neuronal differentiation was induced by RA withdrawal. All experiments were done at 4–6 days after dissociation of EBs.

### Construction of stable mES cells

pcDNA6/V5-His vectors containing cDNAs for flag-tagged ERα, flag-tagged ERβ [42] and LacZ were transfected into undifferentiated mES cells using the mouse neural stem cell nucleofector kit (Amaxa Inc.). Twelve mES clones for each cell line were selected with 10 µg/ml blasticidin. The mES clones with the highest ERα, ERβ or LacZ expression were used for the studies.

### GT1-1 cell culture and infection

GT1-1 cells (passages 14–23) were cultured in 10 cm culture plates with DMEM supplemented with 5% FBS, 5% horse serum (Hyclone) and 2 mM glutamine. GT1-1 cells were plated at 300,000 cells/well on 25 mm glass coverslips coated with Matrigel (BD Biosciences). When the cells reached 60–70% confluency, they were infected for 2 h with 5 or 10 MOI (multiplicity of infection) of adenoviruses expressing ERα, ERβ or LacZ (Viraquest). Viruses were removed after 2 h and the cells were incubated for 24 h after infection. The medium was then replaced with Opti-MEM and incubated for an additional 24 h before testing.

### Adenoviral siRNA constructs

Adenoviruses delivered A-kinase anchoring protein 150 (AKAP150) siRNA (Ad-si-AKAP) or luciferase siRNA (Ad-si-Luc) were prepared as previously described [43]. Viruses were produced by transfecting adenoviral DNA into HEK293 cells, purified on cesium chloride gradients and titered using Adenoviral Rapid Titer Kit (Clontech). Viral titers were 2.8×10^9^ pfu/ml for Ad-si-AKAP, and 2.3×10^9^ pfu/ml for Ad-si-Luc.

### Immunocytochemistry

Cells were fixed with 4% paraformaldehyde, permeabilized with 0.3% triton X-100, blocked and incubated with primary antibodies at 4°C overnight. The antibodies against choline acetyltransferase (ChAT, 1∶100), GABA (1∶1000), glial fibrillary acidic protein (GFAP, 1∶1000), glutamate decarboxylase-67 (Gad67, 1∶500), microtubule-associated protein-2 (Map2, 1∶400), neuronal nuclei (NeuN, 1∶50), oligodendrocytes-O4 (O_4_, 1∶100), serotonin (1∶200), synaptophysin (Syp, 1∶200), Tau (1∶200), βIII tubulin (TujIII, 1∶100), vesicular glutamate transporter-2 (VGlut, 1∶100) and tyrosine hydroxylase (TH, 1∶100) were purchased from Millipore. The antibodies against L-type voltage-dependent calcium channel alpha 1C subunit (CaV1.2, 1∶100) and AKAP150 (1∶100) were purchased from Santa Cruz Biotech Inc. The antibodies against flag (1∶200) and Gad65 (1∶100) were obtained from Sigma-Aldrich. Subsequently, the cells were stained with appropriate Alexa fluor-488 or -594 conjugated secondary antibodies (Invitrogen) and nuclei were stained with DAPI. Images were photographed with a Leica fluorescence microscope.

### RNA extraction and real-time RT-PCR

Total RNA was extracted with Aurum total RNA mini kit (Bio-Rad) and cDNA synthesis was performed with the iScript cDNA synthesis kit (Bio-Rad). The sequence of the primers for GAPDH, ERα and ERβ were reported previously [42] or obtained from Primer Bank (http://pga.mgh.harvard.edu/primerbank/index.html). Real-time PCR analysis was performed in duplicate using iQSYBR Green Mix with an iCycler thermal cycler. The data were analyzed using comparative threshold cycle method using GAPDH as internal control.

### Immunoprecipitation and western blots

Cells were lysed in radioimmunoprecipitation assay buffer (1% Igepal CA-630, 0.5% sodium deoxycholate, 0.1% SDS). Immunoprecipitates were prepared by incubating the lysates with polyclonal anti-ERα (sc-7207, Santa Cruz Biotech. Inc.), polyclonal anti-ERβ (sc-6821, Santa Cruz Biotech. Inc.), monoclonal anti-ERβ (Ab17, raised against full-length human ERβ fused to maltose binding protein) or monoclonal anti-flag (Sigma-Aldrich) at 4°C overnight, followed by incubating with protein-G or protein-A agarose beads (Invitrogen) at 4°C for 6 hours. The immunoprecipitates were separated by SDS-PAGE, transferred to a polyvinylidene fluoride membrane and probed with monoclonal anti-ERα (M7047, DakoCytomation), anti-ERβ or anti-flag. Cell lysates for immunoblotting were prepared by direct lysis of cells in 1× sodium dodecyl sulfate sample buffer. The samples were immediately scraped off, sonicated and heated. Equal amount of lysates were loaded (20 µg of total protein per lane), separated by sodium dodecyl sulfate polyacrylamide gel electrophoresis and transferred to a polyvinylidene fluoride membrane. The following primary antibodies were purchased from Cell Signaling Technologies and used at 1∶1000 dilutions: phospho-(Thr638/641) PKC-α/βII (no. 9375), PKC-ζ (no. 9368), phospho-(Ser473) AKT (no. 9271, 4060), AKT (no. 9272), phospho-(Ser259) c-RAF (no. 9421), c-RAF (no. 9422), phospho-(Thr202/Tyr204) ERK1/2 (no. 4370), ERK1/2 (no. 4695), phospho (Ser133) CREB (no. 9191), CREB (no. 9197). The blots were then incubated with corresponding secondary antibodies conjugated to alkaline phosphatase. The signals were visualized by incubating the membranes with a 5-bromo-4-chloro-3-indolylphosphate/nitroblue tetrazolium solution (Roche). The bands were scanned and quantified with ImageJ (National Institute of Health).

### Calcium assays

Intracellular calcium concentration was measured using fluorescence ratio imaging with MetaFluor Imaging Software (Universal Imaging Corp.) as previously described [44]. Briefly, neurons derived from mES or hES cells, or GT1-1 cells were cultured on coated glass coverslips and loaded with 5 µM Fura-2AM (Invitrogen) for 30 min at 37°C in oxygenated Locke's medium supplemented with 0.1% BSA. The cells were then washed in fresh Locke's medium for 10 min. Coverslips were placed in a temperature controlled modified Sykes-Moore chamber mounted on a Nikon TE2000 inverted fluorescence microscope. Cells were immersed in 300 µl of Locke's medium, and drug treatments were added directly into the medium. Fura-2 fluorescence was measured at wideband emission setting of 510 nm at 5 sec intervals for 10 min of baseline recording and then 10 min after exposure to drugs at excitation settings of 340 and 380 nm. Approximately 40–100 cells were imaged per coverslip.

### Quantification of calcium oscillations

A standard curve was generated using a Fura-2 Ca2+ calibration kit (Invitrogen) where images are acquired at known Ca2+ concentrations. The intracellular calcium concentration ([Ca2+]i) was estimated from the ratio of the emissions and comparison with Fura-2 standards [45]. We programmed Java interface for Pulsar [46], [47] to detect peaks in [Ca2+]i oscillations. The standard deviation (SD) was determined by using the mean of the SD identified by time series analysis using a 5 sec window as described [48], [49]. The coefficient of variation for our calcium imaging was described by the formula: Y  =  4.1827X + 0.4826. The peak detection parameters for Pulsar, G(1)-G(5), were 1.77, 1.05, 0.73, 0.53 and 0.39. A smoothing window of 25 sec was used to determine segmented baseline values. The frequency (numbers per 10 min) of [Ca2+]i peaks during treatment period is calculated as the percentage of the basal levels, which are normalized to 100%. A cell was counted as an excited cell if its frequency of [Ca2+]i peaks was increased at least 50% after treatment in comparison to the basal level. This level was chosen because it was 5 fold of SEM. The time points of “detected peaks” in each cell were recorded as a series of “1”s, and the time points of “no detected peaks” in each cell were recorded as a series of “0”s with a reprogrammed plug-in module of Pulsar. Synchronization of [Ca2+]i peaks among multiple cells was analyzed by random sample permutations using a script in Matlab. As the frequency of [Ca2+]i peaks increase chances that two or more peaks accidentally meet together will increase. To exclude the possibility of accidental encounter of peaks (random synchronization) different cutoffs of synchronization were calculated for the control and treatment period of every coverslip. We defined that a synchronization happened when the number of cells showed [Ca2+]i peaks during a 5 sec interval reached a cutoff level that the possibility of random synchronization was lower than 0.01. The cutoff level was determined by random permutation of the time points of all [Ca2+]i peaks of each cell 2000 times with a time resolution of 5 sec. The peaks were sorted by synchronization levels, i.e., the number of cells with [Ca2+]i peaks occurred at the same 5 sec interval. The minimal number of cells within a 5 sec interval during the 1st percentile of highest synchronized peaks was set as the cutoff level. The 5 sec intervals were chosen since the duration of intracellular calcium peaks has been reported to be greater than 20 sec based on data collected at 1 sec [50], 5 sec [48], and 10 sec [49] intervals in neurons. In addition, recordings taken in 2–10 sec intervals revealed that intervals of less than 5 sec resulted in photobleaching of the Fura-2 dye during the recording period. Comparable results of synchronizations were obtained by Wavelet and random sample permutations. The data calculated by random sample permutations are illustrated in Figure S1.

### Statistics

Statistically significant difference between treatment and control groups was determined by one-way ANOVA, followed by Dunnett's many-to-one comparison procedure and single step method for adjusting p-values in multiple testing with bioconductor package multicomp. The sample size and adjusted p-values are summarized in supplementary Table S1 and S2. Statistical analysis of the frequency of [Ca2+]i peaks was done with raw data. All values of calcium imaging analysis are expressed as means ± SEM. p<0.05 is considered as minimum confidence level.

## Results

### Neurons derived from human ES cells express neuronal markers

**Figure 1:**
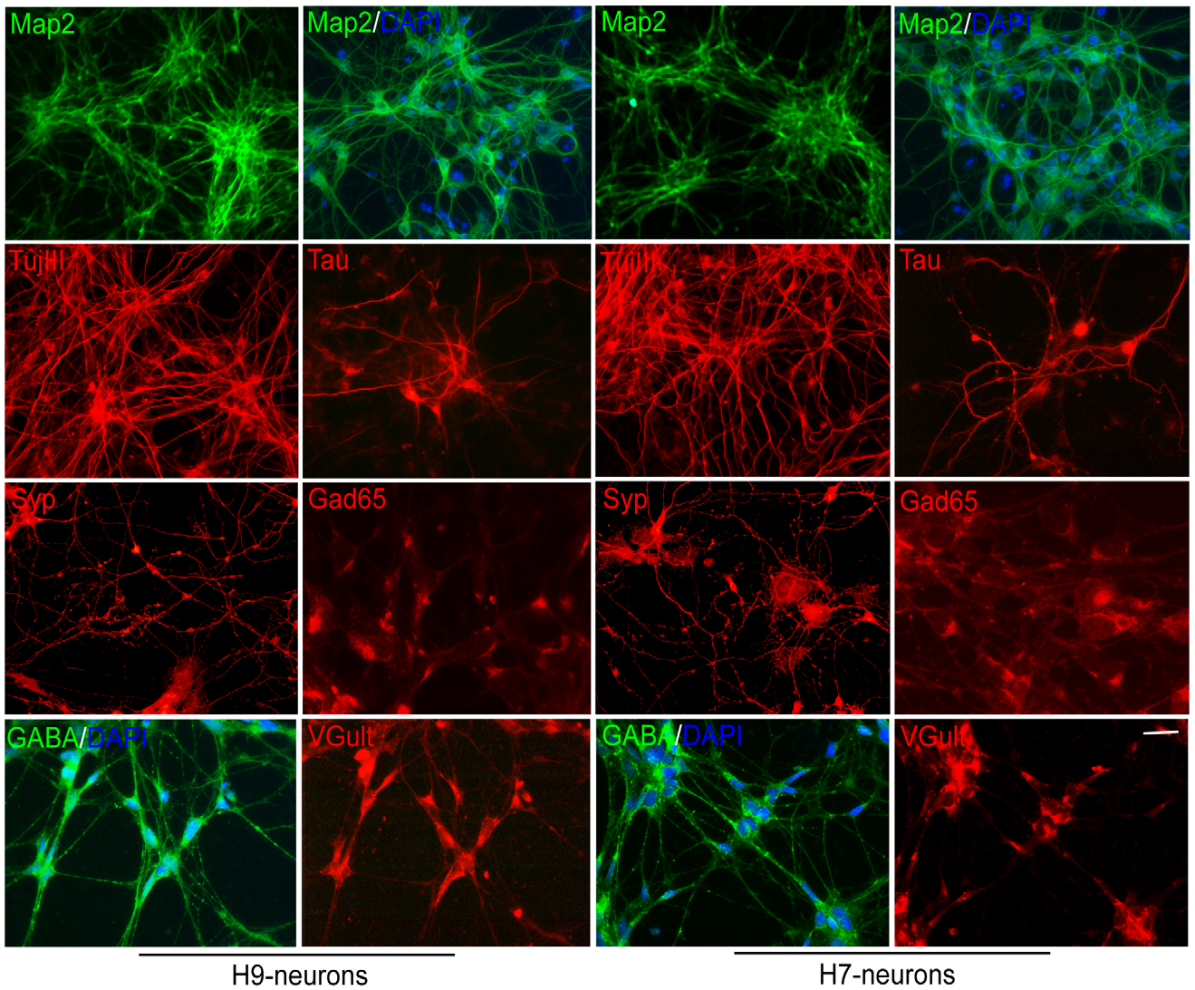
Immunofluorescent staining of neurons from human ES cells with neuronal markers. The cells were stained positively with mature neuronal markers, such as Map2 (green), TujIII (red), Tau (red) and Syp (red) at day 30–36. The homogeneity of culture was shown by 4′,6-diamidino-2-phenylindole (DAPI, blue) and Map2 double-staining. The cells exhibited both GABAergic and glutamatergic properties as shown by glutamic acid decarboxylase (Gad65, red), GABA (green) and vesicular glutamate transporter (VGlut, red) immunofluorescence. The left two lanes are staining of neurons from the H9 cell line, and the right two lanes are staining of neurons from the H7 cell line as labeled. Scale bar: 200 µm for Map2 in row 1, and 63 µm for the rest.

Because neurons derived from other ES cell lines were reported to exhibit distinct differentiation patterns [41], we studied two well established human ES cell lines (H9 and H7). 30–36 days after dissociation of neurospheres, 80–90% of the cells derived from H9 or H7 cells were demonstrated to be neurons by immunofluorescent staining with the mature neuronal markers: Map2, TujIII, Tau and synaptophysin (Syp) (Figure 1). The differentiated neurons expressed subunits for voltage-gated Na+, K+ and Ca2+ channels by microarray analysis (data not shown), indicating that the cells contain ion channels characteristic of neurons. GABAergic (Gad65, GABA) or glutamatergic (VGlut) markers were detected separately in most cells (Figure 1), whereas dopaminergic (TH), cholinergic (ChAT) and serotoninergic (serotonin) markers were not detected (data not shown). The observation that glial markers were absent by microarray analysis (data not shown) indicates that few glial cells or oligodendrocytes were present in the neuronal cultures. Our results demonstrate that the hES cells were efficiently differentiated into GABAergic and glutamatergic neurons that express ion channels characteristic of neurons.

### Neurons derived from human ES cells are excitable

**Figure 2:**
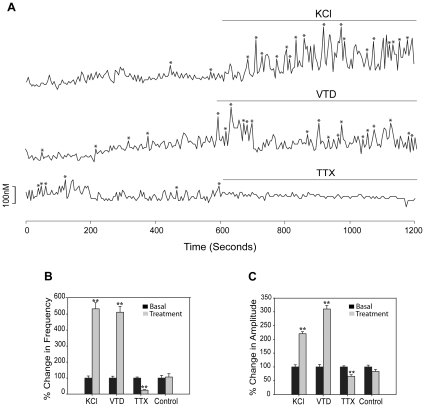
Neurons from the H9 cell line are excitable by ion channel regulators at day 30–36. (A) Tracings of [Ca2+]i changes in response to KCl (56 mM), VTD (50 µM) and TTX (1 µM) in representative cells. “*” indicates Pulsar recognized [Ca2+]i peaks. Changes after treatment (gray bars) in the frequency of [Ca2+]i oscillations (B) and amplitudes of [Ca2+]i peaks (C) compared to basal activity (black bars, normalized to 1). The number of cells analyzed for KCl, VTD, TTX and Control were 180, 175, 104 and 98, respectively, from 2 or 3 independent experiments. ** p<0.01 compared to control.

**Figure 3:**
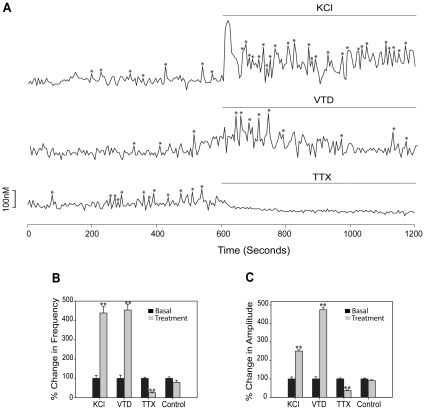
Neurons from the H7 cell line are excitable by ion channel regulators at day 30–36. (A) Tracings of [Ca2+]i changes in response to KCl (56 mM), VTD (50 µM) and TTX (1 µM) in representative cells. “*” indicates Pulsar recognized [Ca2+]i peaks. Changes after treatment (gray bars) in the frequency of [Ca2+]i oscillations (B) and amplitudes of [Ca2+]i peaks (C) compared to basal activity (black bars, normalized to 1). The number of cells analyzed for KCl, VTD, TTX and Control were 164, 189, 172 and 153, respectively from 2 or 3 independent experiments. ** p<0.01 compared to control.

We examined the differentiated neurons for spontaneous or evoked calcium oscillations by using calcium imaging analysis 30–36 days after neurosphere dissociation. Neurons derived from human H9 ES cells were excitable with KCl and veratridine (VTD) (Figure 2A), which cause depolarization by increasing K+ or Na+ inward current. [Ca2+]i oscillations were inhibited by tetrodotoxin (TTX) (Figure 2A), which is a specific blocker of the voltage-gated Na+ channels. The frequency of [Ca2+]i oscillations was increased 5.3-fold by KCl, 5.1-fold by VTD and blocked by TTX (Figure 2B) with the corresponding changes of the amplitudes (Figure 2C) and synchronizations of [Ca2+]i peaks (Table 1). KCl, VTD and TTX produced similar changes in basal [Ca2+]i oscillations (Figure 3A) as well as frequency (Figure 3B), amplitudes (Figure 3C) and synchronizations (Table 1) of [Ca2+]i peaks in neurons derived from human H7 ES cells, demonstrating that both cell lines produce neurons that have similar properties.

**Table 1:** Effect of drugs on the synchronizations of calcium oscillations in neurons from hES cells.

H9-neurons				H7-neurons		
Treatments	Experiments	Cells	No. in 10 min after exposure	Experiments	Cells	No. in 10 min after exposure
KCl	2	180	2.86±0.41	2	164	2.30±0.08
VTD	2	175	3.14±0.62	2	189	2.70±0.24
TTX	2	104	0.28±0.08^a^	2	172	0.48±0.07
Control	2	98	1.28±0.42	3	153	0.87±0.09
E_2_	2	143	1.71±0.09	3	200	2.29±0.27
ERB-041	3	221	2.38±0.45	3	152	1.62±0.23
DPN	3	260	2.16±0.48	3	174	1.28±0.22
MF101	3	188	1.45±0.29	3	156	1.32±0.35
PPT	2	134	1.09±0.47	3	202	0.94±0.20
PPT+ERα	3	179	0.98±0.16	3	197	0.98±0.34
Control	2	98	1.28±0.42	3	153	0.87±0.09

a p<0.05 vs. respective control.

### ERβ agonists stimulate [Ca2+]i oscillations in neurons derived from human ES cells

**Figure 4:**
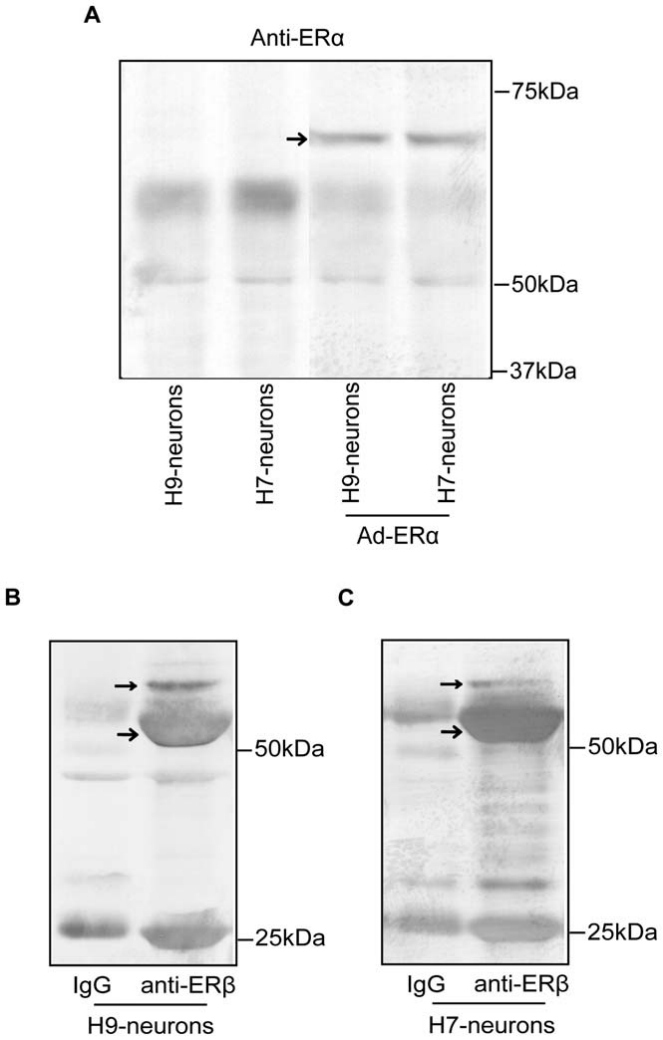
Neurons from the human ES cell lines express endogenous ERβ. (A) Immunoprecipitation and western blot analysis with anti-ERα of H9- and H7-neurons. No endogenous ERα expression was revealed in both neurons (left two lanes). Exogenous ERα (pointed by arrows) was introduced by infection with 10 MOI of Ad-ERα (right two lanes). The blots have been rearranged from the same gel. (B) Immunoprecipitation and western blot analysis with anti-ERβ of H9- (left panel) and H7-neurons (right panel). The usual size ERβ (denoted by →) and a slightly bigger ERβ isoform (denoted by →) were detected in both H9- and H7-neurons. Immunoprecipitation with IgG from the same amount of cell lysates was used as negative control.

**Figure 5:**
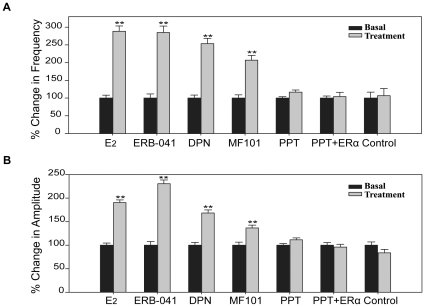
ERβ-selective ligands increase [Ca2+]i oscillations in H9-neurons. Changes in the frequency of [Ca2+]i oscillations (A) and amplitudes of [Ca2+]i peaks (B) after treatment with E_2_ (10 nM), ERB-041 (1 µM), DPN (1 µM), MF101 (125 µg/ml), PPT (1 µM), PPT plus Ad-ERα (PPT+ERα) and Control. The data after treatment (gray bars) are expressed relative to those before treatment (basal, black bars), which are normalized to 1. The number of cells analyzed for the above groups were 143, 221, 260, 188, 134, 179 and 98, respectively from 2 or 3 independent experiments. ** p<0.01, H9-neurons treated by different drugs are compared to control.

Our data indicated that neurons derived from both H9 and H7 cell lines have the morphological and physiological properties of mature neurons. These results indicate that these cells are a potential cell based system to study the action of estrogens if they express ERs. No ERα was detected in neurons derived from H7 or H9 cells unless they were infected with Ad-ERα (Figure 4A). Immunoprecipitation and western blot analysis with antibodies to ERβ show that the neurons derived from H9 (Figure 4B) and H7 (Figure 4C) cells expressed ERβ with a MW of 55 kDa. A small band with a MW of 59–60 kDa which is likely an ERβ isoform was also present in the neurons. There is also a band at 25 kDa (Figure 4B, Figure 4C), which likely represents a degraded fragment from ERβ. To test if the ERs were functional, the neurons were treated with E_2_ and calcium parameters were measured. In neurons from H9 cells, E_2_ produced a 2.9-fold increase of the frequency of [Ca2+]i oscillations (Figure 5A, Figure S2), a 1.9-fold increase of the amplitudes (Figure 5B) and a 1.7-fold increase of the synchronizations (Table 1) of [Ca2+]i peaks within a few minutes. Similar results were observed in neurons from the H7 cell line (Figure S3, Figure S4). To determine which ER subtype was responsible for mediating the rapid stimulatory effect of E_2_ on [Ca2+]i oscillations, the cells were treated with ERα or ERβ-selective agonists. The ERα selective agonist, PPT did not produce any significant changes in calcium influx (Figure 5A, Figure 5B, Figure S2, Table 1). Furthermore, there was no change in [Ca2+]i oscillations or amplitudes after treatment with PPT (Figure 5A, Figure 5B, Table 1) in neurons from the H9 and H7 cells infected with an adenovirus that expresses ERα (Figure 4A). The three ERβ agonists, ERB-041, DPN and MF101 produced an increased frequency of [Ca2+]i oscillations, amplitudes and synchronizations of [Ca2+]i peaks (Figure 5A, Figure 5B and Figure S2, Table 1). Similar results were observed in neurons from the H7 cell line (Figure S3, Figure S4). These results demonstrate that ERβ mediates the stimulatory effects on [Ca2+]i oscillations in neurons from hES cells.

### Neurons from mouse ES express neuronal markers and are excitable

To begin to explore the mechanism whereby ERβ agonists increase [Ca2+]i oscillations in neurons, we used neurons from mES cells because they can be obtained in greater abundance and purity compared to hES cells. Five days after dissociation of EBs, about 90–95% of the cells were neurons as determined by double staining with DAPI and the neuronal markers: Map2, TujIII, NeuN and Tau (Figure S5). A homogenous appearance (similar shape and size) of neuronal cell bodies was also observed by NeuN staining. Synapse development in the culture was displayed by staining with Syp along neuronal processes (Figure S5). The cells expressed subunits of voltage-gated Na+, K+ and Ca2+ channels by microarray and quantitative PCR analysis (data not shown). Furthermore, the cells positively stained with CaV1.2, which is a pore-forming subunit of the L-type voltage-gated Ca2+ channel (VGCC) (Figure S5). Similar to neurons derived from hES cells, the mouse neurons expressed Gad67, GABA and VGlut in most cells (Figure S5), indicating that the culture contained both GABAergic and glutamatergic neurons. Within the first week, less than 1% cells were positive for glial cell markers (GFAP, O_4_) (data not shown). The cultures were negative for dopaminergic (TH), cholinergic (ChAT) and serotoninergic (serotonin) markers (data not shown). The majority of neurons demonstrated spontaneous or evoked calcium oscillations as detected by calcium imaging analysis at 4–7 d after dissociation of EBs. The excitability of cells was increased by KCl and VTD, and decreased by TTX (Figure S6A). Consistent with changes of the frequency of [Ca2+]i oscillations (Figure S6B), the amplitude of [Ca2+]i peaks (Figure S6C) and the frequency of synchronized events (Table 2) were increased by KCl or VTD, and decreased by TTX. These data demonstrate that neurons derived from mES cells have the morphological and physiological features characteristic of mature GABAergic and glutamatergic neurons with similar properties as those derived from hES cells.

**Table 2:** Effect of drugs on the synchronizations of calcium oscillations in neurons from mES cells.

Figures	Treatments	Experiments	Cells	No. in 10 min after exposure
Figure 7	Nif	5	233	0.44±0.15
	Nif+E_2_	4	213	1.07±0.16
	AgTx	3	163	0.65±0.12
	AgTx+E_2_	3	170	1.28±0.07
	CgTx	3	165	0.44±0.11
	CgTx+E_2_	3	156	1.17±0.19
	E_2_-BSA	3	139	2.17±0.56
	E_2_	3	113	2.38±0.33
	Control	2	99	0.78±0.13
Figure 8	Ad-si-AKAP	7	366	1.16±0.11
	Ad-si-Luc	6	286	5.14±1.28^b^
	Control	6	320	0.99±0.15
FigureS6	KCl	3	164	2.14±0.43
	VTD	3	156	2.51±0.28
	TTX	4	196	0.22±0.04^b^
	Control	2	111	1.17±0.28
FigureS7	E_2_-Ca^2+^	3	140	1.59±0.86
	E_2_+Ca^2+^	3	140	5.47±1.74
FigureS10	E_2_	8	307	2.12±0.27
	ERB-041	8	331	1.62±0.23
	DPN	6	337	1.69±0.15
	MF101	6	330	1.79±0.19
	PPT	6	296	0.84±0.14
	PPT_10uM_	5	270	0.90±0.14
	PPT+ERα	6	288	1.07±0.19
	E_2_+ERβ	6	313	2.01±0.41
	Control	2	110	0.95±0.10

b p<0.01 vs. respective control.

### Neurons derived from mouse ES cells express endogenous ERα and ERβ

**Figure 6:**
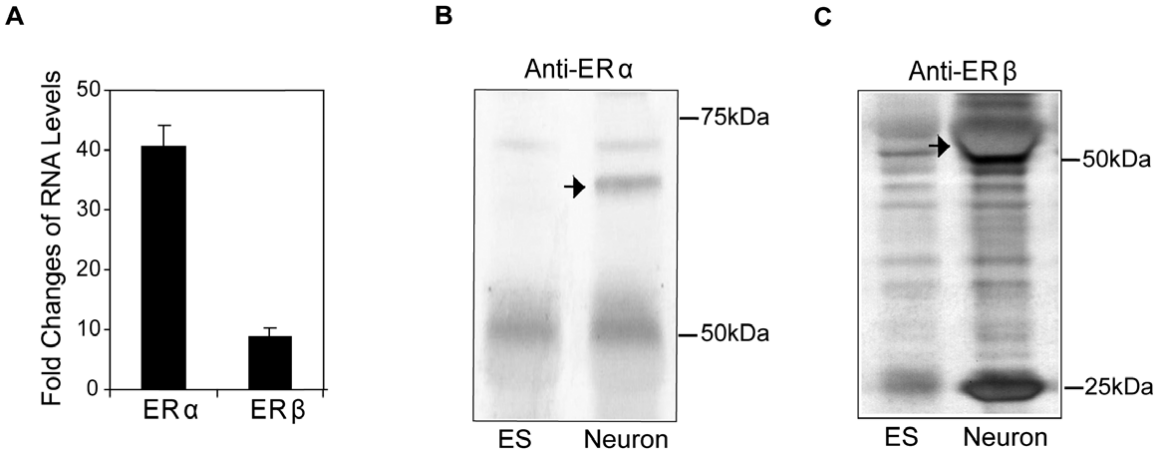
Neurons from mouse ES cells express endogenous ERα and ERβ. (A) Real-time PCR analysis of ERα and ERβ mRNAs in neurons relative to undifferentiated mES cells (assigned to 1). The data shown are the average of ten independent cultures. Immunoprecipitation and western blot analysis of ERα (B) and ERβ (C) (pointed by arrows) in neurons (Neuron) and undifferentiated ES14 cells (ESC).

We used quantitative real-time PCR to determine if ERα or ERβ mRNA were expressed in neurons from mES-cells. The expression of ERα and ERβ increased in parallel with neuronal markers, peaked at 4 days and remained at high levels through 7 days after dissociation of EBs (data not shown). A 40.7-fold increase of ERα and an 8.9-fold increase of ERβ mRNA levels were observed 4–6 days after dissociation (Figure 6A). The presence of ERα (Figure 6B) and ERβ (Figure 6C) proteins were confirmed by immunoprecipitation and western blot analysis. Our findings demonstrate that neurons from mES cells contain endogenous ERs, and therefore can be used as a cell model system to study the mechanisms of estrogenic compounds in neurons.

### E_2_ induced extracellular Ca2+ influx through L-type VGCC

**Figure 7:**
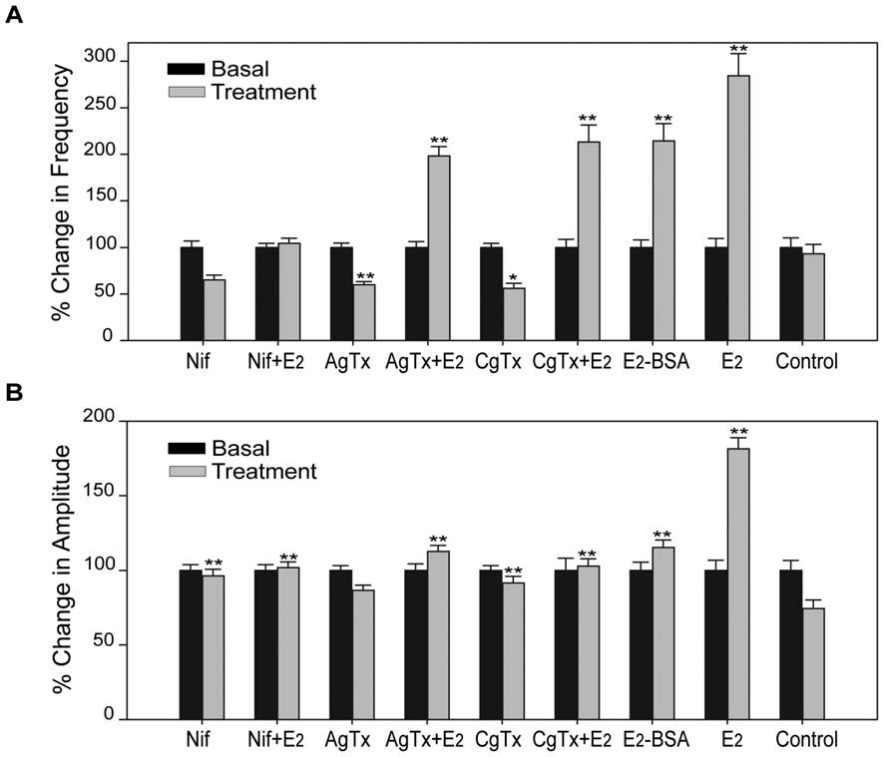
E_2_-mediated increase of [Ca2+]i oscillations occurs through L-type Ca2+ channels. Neurons from mES cells were pretreated for 15 min with different Ca2+ channel inhibitors: nifedipine (Nif, 10 µM), ω-conotoxin GVIA (CgTx, 1 µM), ω-agatoxin IVA (AgTx, 200 nM) before exposed to 10 nM E_2_. Alternatively, the cells were treated directly with the above inhibitors, E_2_ and membrane-impermeable E_2_-BSA (10 nM). Changes after drug treatment (gray bars) in the frequency of [Ca2+]i oscillations (A) and amplitudes of [Ca2+]i peaks (B) compared to basal levels (black bars, normalized to 1). The number of cells for Nif, Nif+E_2_, AgTx, AgTx+E_2_, CgTx, CgTx+E_2_, E_2_-BSA, E_2_ and Ctrl are 233, 213, 163, 170, 165, 156, 139, 113 and 99, respectively, from 5, 4, 3, 3, 3, 3, 3, 3, and 2 independent experiments. ** p<0.01, * p<0.05 in comparison to non-treatment control.

To determine whether neurons from mES cells were responsive to estrogens in a similar manner to those from hES cells they were treated with E_2_. A 3.1-fold increased frequency of [Ca2+]i oscillations was observed in the neurons (Figure S10A) within 1 min following E_2_ treatment. Depletion of extracellular Ca2+ from perfusion buffer completely abolished E_2_ potentiation of [Ca2+]i oscillations, whereas adding back Ca2+ into the medium restored the effects (Figure S7A). All parameters of [Ca2+]i oscillations, including the frequency (Figure S7B), the percentage of responsive cells (Figure S7C) and amplitude (Figure S7D) of [Ca2+]i peaks, and synchronized events (Table 2) were markedly increased after addition of Ca2+. Thus, the E_2_-induced rise of [Ca2+]i oscillations depends on extracellular Ca2+. We sought to determine which subtype of voltage-gated calcium channel (VGCC) is modulated by E_2_. The cells were pretreated for 15 min with specific calcium channel blockers, including nifedipine (L-type), conotoxin (N-type) and agatoxin (P-type) prior to exposure to E_2_. Approximately 30–40% of baseline frequency (Figure 7A) and amplitudes (Figure 7B) of [Ca2+]i oscillations were decreased by exposure to each inhibitor alone (Table 2), suggesting that all three types of VGCC were expressed in neurons from mES cells. However, only nifedipine blocked the E_2_-mediated increase in the frequency of calcium oscillations (Figure 7A, Figure S8), amplitudes (Figure 7B) and synchronizations (Table 2). These findings indicate that the E_2_-mediated rapid Ca2+ influx occurs through L-type VGCC. The membrane impermeable E_2_-BSA was found to act similarly to E_2_, but it was less potent (Figure 7A, Figure 7B, Table 2). This observation indicates that the effects of E_2_ are mediated by plasma membrane ERs, rather than intracellular ERs.

### E_2_ potentiation of [Ca2+]i oscillations requires ERβ

Similar to the data obtained in neurons from hES cell lines, the non-selective ER agonist E_2_ and three distinct ERβ-selective agonists, ERB-041, DPN and MF101 increased [Ca2+]i oscillations, amplitudes and synchronizations in neurons from mES cells, whereas ERα-selective agonist PPT did not produce any changes of [Ca2+]i peaks (Figure S9, Figure S10A, Figure S10B, Table 2). To address the unlikely possibility that the cells have insufficient amount of ERα to produce a response, we established a stable mES cell line that overexpresses a flag-tagged human ERα, which is known to be functional in other cell types [42]. The presence of flag-ERα in undifferentiated mES cells and neurons was shown by nuclear staining with anti-flag (Figure S11A). Immunoprecipitation and western blot analysis with antibodies to the flag peptide and ERα confirmed the existence of flag-ERα (Figure S11B). Even with the overexpression of ERα there was no increase in the frequency (Figure S10A) of [Ca2+]i oscillations, amplitudes (Figure S10B) or synchronizations (Table 2) after PPT treatment. A stable mES cell line that overexpresses a flag-tagged human ERβ [42] was also prepared (Figure S11C, Figure S11D). A similar response to E_2_ was obtained in mES cells overexpressing flag-ERβ as wild-type mES cells (Figure S10A, Figure S10B, Table 2).

### E_2_ increased [Ca2+]i oscillations in GT1-1 cells infected with ERβ adenovirus

It has been shown that estrogens can produce nongenomic effect though proteins other than ERα or ERβ, such as ER-X [51], GPR30 [52] and Gq-mER [53]. These studies suggest the effects of estrogens might be mediated through other estrogen binding proteins, rather than ERβ, even though all three ERβ agonists increased calcium oscillations. To further characterize the role of ERβ on calcium oscillations, we used the immortalized GT1-1 cells. Because these cells did not express detectable levels of ERβ, they were infected with an adenovirus that expresses ERβ. A 7.2-fold increase of ERβ mRNA occurred in cells infected with 5 MOI of the ERβ adenovirus (Figure S12A). The addition of E_2_ to GT1-1 cells expressing ERβ resulted in a 2.5-fold increased number of [Ca2+]i oscillations, a 1.6-fold increased frequency (Figure S12B) and amplitudes (Figure S12C) and a 2.1-fold increase of the synchronization frequencies (Table 3). No significant changes in [Ca2+]i oscillations were observed in GT1-1 cells expressing ERα or a LacZ control adenovirus (Figure S12B, Figure S12C, Table 3). These data provide further evidence that the effects of E_2_ on neuronal [Ca2+]i oscillations are mediated through ERβ signaling.

**Table 3:** Effect of drugs on the synchronizations of calcium oscillations in GT1-1 neurons.

Treatments	Experiments	Cells	No. in 10 min after exposure
Ad-ERα	5	174	0.98±0.15
Ad-ERβ	5	164	2.10±0.19^a^
Ad-LacZ	4	149	0.88±0.08

a p<0.05 vs. Ad-LacZ.

### AKAP150 knockdown reduces [Ca2+]i oscillations induced by E_2_

**Figure 8:**
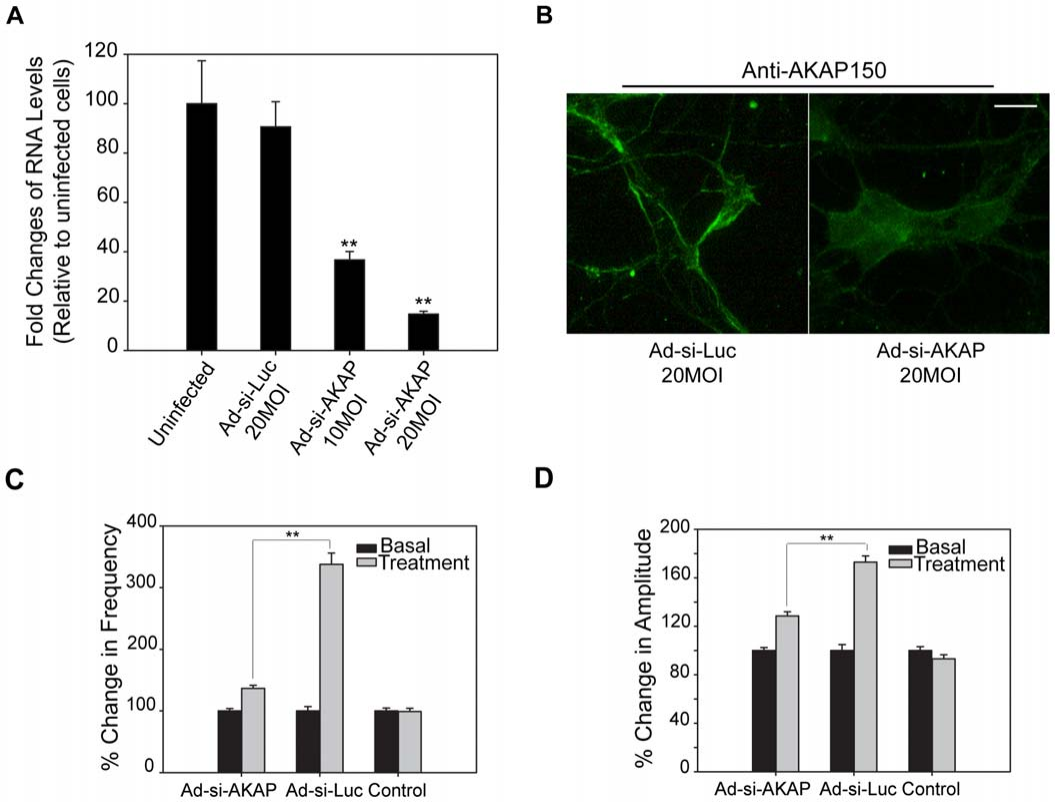
AKAP150 knockdown decreases [Ca2+]i oscillations induced by E_2_. (A) RNA levels of AKAP150 in neurons from mES cells at 48 h postinfection with adenovirus delivering siRNA to AKAP150 (Ad-si-AKAP) and luciferase (Ad-si-Luc). The data shown are normalized to AKAP150 levels of uninfected cells (assigned to 1), and are the average of three independent experiments. (B) Immunofluorescent staining of AKAP150 (green) in mES-neurons at 48 h postinfection with 20 MOI Ad-si-AKAP or Ad-si-Luc. Scale bar represents 63 µm. Changes in the frequency (C) and amplitudes (D) of [Ca2+]i peaks after E_2_ (10 nM) treatment in neurons from mES cells at 48 h postinfection with 10 MOI of Ad-si-AKAP, Ad-si-Luc and uninfected control (Ctrl). The data after treatment (gray bars) are expressed relative to basal levels (black bars, normalized to 1). The numbers of cells analyzed for the above groups are 366, 286, 320, respectively, from 6 or 7 independent experiments. **p<0.01, Ad-si-AKAP cells are compared to Ad-si-Luc cells.

We have shown that E_2_ increases [Ca2+]i oscillations through L-type calcium channels (Figure 7). The A kinase anchoring protein 150 (AKAP150) is critical for regulating CaV1.2 (L-type) voltage-gated Ca2+ channels in cardiac myocytes [54], [55] and neurons [56]. Our observation that CaV1.2 is highly expressed in neurons from mES cells (Figure S5), suggests that AKAP150 might be involved in signaling pathway of estrogens. To investigate the role of AKAP150 in E_2_-mediated Ca2+ influx, we used an AKAP-siRNA adenovirus (Ad-si-AKAP) to knockdown AKAP150 in neurons, and a luciferase siRNA adenovirus (Ad-si-Luc) as a toxicity and specificity control. A dose-dependent knockdown of AKAP150 mRNA was observed with 10 or 20 MOI of Ad-si-AKAP (Figure 8A). A reduction of AKAP150 protein by Ad-si-AKAP was confirmed by weaker staining with anti-AKAP150 along neurites and membrane in the neurons (Figure 8B). The increased frequency (Figure 8C), amplitudes (Figure 8D) and synchronizations (Table 2) of [Ca2+]i oscillations by E_2_ were significantly attenuated in neurons infected with Ad-si-AKAP in comparison to those infected with Ad-si-Luc. These results indicate that AKAP150 participates in E_2_-induced Ca2+ influx through L-type calcium channels.

### Ca2+-dependency of rapid activation of multiple signaling pathways by E_2_

**Figure 9:**
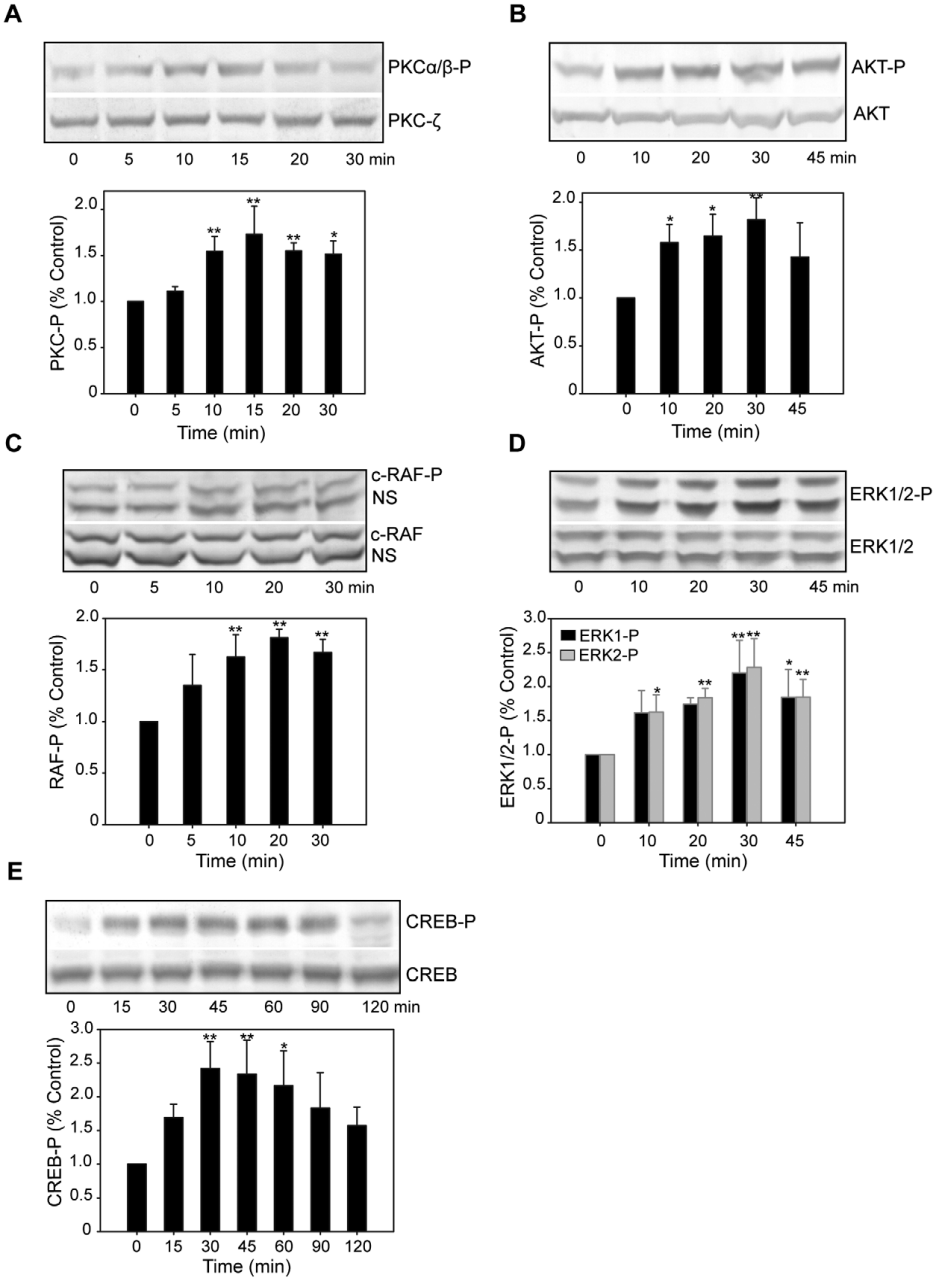
E_2_ rapidly activates multiple signaling transduction events in neurons from mES cells. The cells were treated with E_2_ (10 nM), lysed at the indicated time points and subjected to SDS-PAGE. Western blotting was performed with phospho-specific antibodies against PKCα/β-P (A), AKT-P (B), c-RAF-P (C), ERK1/2-P (D) and CREB-P (E), or with corresponding phospho-independent antibodies against total PKC (PKC- ζ), AKT, c-RAF, ERK1/2 and CREB as loading controls. A representative immunoblot is shown for each signaling pathway. Densitometric analysis is plotted in columns below the blots. Levels of E_2_-induced activation were calculated as fold-increase in comparison to time point zero (normalized to 1). The results shown are the average ± SD of three independent experiments done in three independent cultures. NS: non-specific band of c-RAF-P or c-RAF. ** p<0.01, * p<0.05, all time points after E_2_ exposure were compared to time point zero.

Our results indicate that estrogens initiate nongenomic actions by causing an influx of calcium through L-type calcium channels. Additionally, estrogens could cause nongenomic effects by activating other signaling pathways, because they have been shown to activate multiple pathways in neurons including the mitogen-activated protein kinase (MAPK)/extracellular signal-regulated kinase (ERK), phosphoinositide 3-kinase (PI3K)/AKT, alpha-Ca2+/calmodulin-dependent kinase II and protein kinase C (PKC) pathways [57], [58], [59], [60]. We studied the activation of multiple signaling transduction cascades after E_2_ treatment in the neurons from mES cells. Using immunoblotting and phospho-specific antibodies, we found that E_2_ produces a rapid, transient phosphorylation of PKC (Figure 9A), AKT (Figure 9B), c-RAF (Figure 9C), ERK1/2 (Figure 9D) and CREB (Figure 9E). Significant phosphorylation of PKC, AKT, c-RAF, ERK1/2 was observed after 10 min of treatment, whereas a significant increase in CREB phosphorylation was observed after 30 min (Supplementary Table S3). Maximal phosphorylation above control levels peaked at between 10–30 min, and then returned to baseline levels within 10–15 min. (Figure 9A, B, C, D and E). The E_2_-induced phosphorylation of AKT and ERK1/2 was inhibited by the PI3K inhibitor LY294,002 and MEK1/2 inhibitor U0126 (data not shown). These results indicate that E_2_-induced activation of AKT and ERK1/2 are mediated through PI3K and MAPK signaling pathways, respectively. Consistent with the data obtained with [Ca2+]i oscillation assay, the activation of signaling networks by E_2_ was abolished by pretreatment with nifedipine (data not shown), and thus is likely mediated through L-type calcium channels.

### E_2_ mediated activation of MAPK and PI3K pathways through ERβ

**Figure 10:**
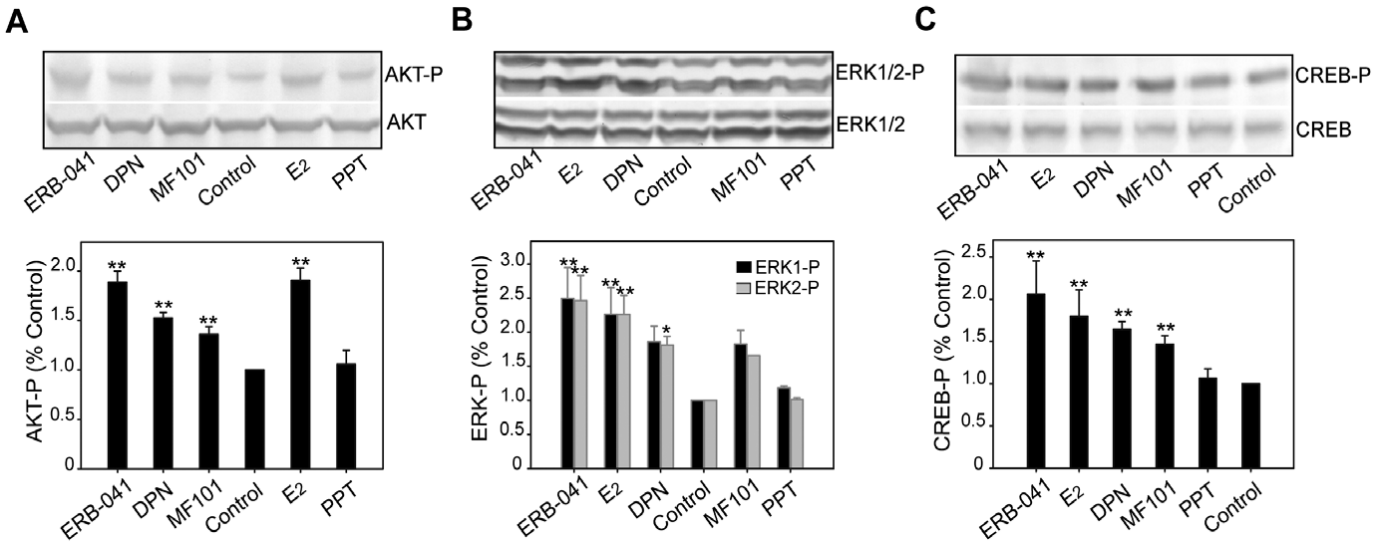
ERβ-selective ligands activate AKT, ERK1/2 and CREB signaling in neurons from mES cells. The cells were treated with E_2_ (10 nM), ERB-041 (1 µM), DPN (1 µM), MF101 (125 µg/ml), and PPT (1 µM) for 30 min, lysed and analyzed by western blotting using phospho-specific AKT (A), ERK1/2 (B) and CREB (C). Total AKT, ERK1/2 and CREB blots were used as loading controls. Blots from a representative experiment were shown. Bands were quantified as fold-increase compared to untreated control (assigned to 1). The average ± SD of 3 or 4 independent experiments were plotted in columns. **p<0.01, * p<0.05, all treatment groups were compared with non-treatment control.

To examine role of ERα and ERβ in mediating rapid signaling by E_2_ in neurons from mES cells, we investigated the phosphorylation of AKT, ERK1/2, and CREB after exposure to ER subtype-selective agonists. ERB-041, DPN and MF101 increased the phosphorylation of AKT (Figure 10A), ERK1/2 (Figure 10B), and CREB (Figure 10C) signaling similar to E_2_, whereas PPT was ineffective. The intensity of activation by different ERβ agonists was consistent with the magnitude of their induction of [Ca2+]i influx. (Supplementary Table S3). Stronger activators of [Ca2+]i oscillation such as E_2_ and ERB-041 induced a 1.8- to 2.5-fold increase above basal levels, and moderate activators such as DPN and MF101 induced a 1.4- to 1.8-fold increase in phosphorylation (Figure 10A, Figure 10B, Figure 10C, Supplementary Table S3).

## Discussion

Primary neuronal cultures have been prepared from embryonic hippocampus or hypothalamus [32], both of which contain ERs to study the neuronal effects of estrogens. Additionally, relatively purified GnRH neurons can be obtained from the embryonic olfactory placode of mice [61] or rhesus monkeys [62], which is the site of origin for GnRH neurons before they migrate to the hypothalamus where they become widely scattered. However, primary neuronal cultures from animals are often mixed with non-neuronal cells and cannot be maintained continuously in culture because they do not proliferate due to the postmitotic property of neurons. Other studies have used the immortalized GT1-1 hypothalamic GnRH-secreting neuronal cell line. This cell line expresses a neuronal-specific phenotype, but its derivation from an SV40 T-antigen induced tumor may be problematic [36]. Our data demonstrated that GT1-1 neurons failed to respond to E_2_. Although ERα and/or ERβ transcripts and proteins have been reported in GT1-1 cells [37], [63], it has been difficult to detect estrogen responses in these cells, probably due to low expression levels of ERs. While studies with these different neuronal models have been valuable to understand how estrogens act on neurons and cause nongenomic effects, it is important to develop other neuronal models that can be maintained continuously *in vitro*, particularly human neurons because of the difficulty in obtaining neurons from human brain tissue. Here we show that both neurons derived from human and mouse ES cells represent an additional model to study the neuronal effects of estrogens. The neurons retain the morphological and electrophysiological properties of mature neurons. Both neurons from hES and mES cells express ERβ, whereas only the neurons from the mES cells expressed ERα. It is likely that neurons from hES cells express ERα, but the levels are too low to detect. We found that the neurons were a very sensitive estrogen responsive system based on changes in [Ca2+]i parameters. E_2_ rapidly increased the frequency and amplitude of [Ca2+]i peaks and the frequency of synchronizations. Once we established that the ES-derived neurons were responsive to the non-selective ER agonist, E_2_, we investigated if the effect on calcium oscillations was mediated by ERα or ERβ using ER-selective ligands. These studies demonstrated that E_2_ increases [Ca2+]i oscillations and synchronizations through ERβ signaling. This was demonstrated by observation that the cells responded to multiple ERβ-selective agonists with different structures in a rapid and direct manner, but failed to respond to the ERα-selective agonist, PPT. Neurons from mES cells overexpressing a functional ERα [42] also did not respond to PPT, which ruled out that the lack of response was due to insufficient ERα levels in these neurons. Other studies using primary neurons from animals have found that ERβ is involved in regulating calcium influxes. PPT and DPN produce a rapid stimulation of intracellular Ca2+ influx [44] in rat hippocampal neurons. A role for ERβ in regulating neuronal activity is also supported by the observations that several ERβ-selective phytoestrogens [64] inhibit glutamate or Aβ1–42 induced toxicity of rat primary hippocampal neurons. Furthermore, DPN rapidly increased the firing rate of GnRH neurons, whereas PPT did not have any effect [33]. These studies and our findings with neurons derived from ES cells indicate that ERβ has an important role in regulating various neuronal processes. However, it is also clear that ERα can produce nongenomic effects on calcium oscillations in neurons derived from brain tissue [32]. It is possible that neurons derived from ES cells have different intrinsic properties than those derived from the brain because these cells are in contact with other cell types, such as astrocytes. In addition to acting directly on neurons estrogens exert rapid effects on calcium responses in astrocytes, suggesting that astrocyte-neuron communication is important for regulating neuronal activity in response to estrogens [65], [66]. Future studies can be done to evaluate the role of astrocytes on calcium oscillations in neurons from ES cells after treatment with ERα and ERβ-selective ligands. It also will be interesting to determine if there is a different response with ERα and ERβ on calcium oscillations in other neuronal subtypes derived from ES cells, such as dopaminergic neurons.

Several receptors that are not part of the nuclear receptor superfamily have been shown to interact with estrogens, suggesting that it is possible they could be involved in the regulation of calcium oscillations in the neurons derived from ES cells. A high affinity receptor termed, ER-X is present in caveolae rafts from developing neocortical neurons and coupled to activation of the MAPK signaling cascade [51]. A novel Gq membrane ER (Gq-mER) coupled to a PLC–PKC–PKA pathways was found in hypothalamic β-endorphin, dopamine, and GABAergic neurons with coupling to µ-opioid and/or GABA receptors [67]. The synthetic nonsteroidal compound, STX binds to Gq-mER and mimics the action of E_2_ in ERα and ERβ knockout mice [68], [69]. Estrogens also bind to another G-protein, G-protein coupled receptor 30 (GPR30), which was first indentified in breast cancer cells [52], [70], [71], [72], and more recently in different regions of the rat brain [73], [74]. We provided evidence from several studies that indicate ERβ actually mediates the changes in [Ca2+]i oscillations in neurons from ES cells, rather than these proteins. First, the increased [Ca2+]i oscillations by E_2_ was reproduced in GT1-1 neurons infected with an adenovirus expressing ERβ but not Ad-ERα or Ad-LacZ. Second, the stimulatory effects of E_2_ on [Ca2+]i oscillations were blocked by tamoxifen (data not shown), which competes with E_2_ for binding to ERα and ERβ [75]. Third, the effect on [Ca2+]i oscillations occurred with three different classes of ERβ agonists, making it unlikely that all three classes will interact these alternative ERs.

ERα and ERβ that are identical to the nuclear ERs have been localized to the plasma membrane and may mediate rapid signaling [76], [77], [78]. Consistent with other studies [79], [80], we found that a BSA-conjugated estradiol stimulated [Ca2+]i oscillations in neurons. These findings indicate that the effect of estrogens on calcium oscillations is mediated by a plasma membrane bound ERβ. Our study demonstrated that ERβ, but not ERα was involved in the stimulation of [Ca2+]i oscillations by estrogens. The mechanisms underlying the differential regulation by ERα and ERβ in neurons from ES cells are unclear. Several possibilities are likely. First, ERα and ERβ may be differentially recruited to plasma membrane after E_2_ exposure, because translocation of ERβ to plasma membrane is observed within 5 min after exposure to E_2_ in hippocampal neurons [81]. Second, ERβ, but not ERα may be coupled to G-proteins or other factors involved in calcium signaling via an unknown mechanism. Third, ERα and ERβ might be coupled to different signaling cascades through the interaction with different cofactors associated with membrane proteins as previously shown in other cell types [38]. We found that ERβ is involved in the activation of the PKC, MAPK and AKT pathways in neurons from mES cells, which is consistent with other studies [58], [82], [83]. Future studies will be needed to determine if ERβ regulates these pathways in neurons derived from human ES cells. The activation of these pathways in neurons from mES cells is dependent on calcium mobilization from L-type VGCC, because nifedipine blocks the increase in calcium oscillations and the phosphorylation of PKC, Raf-1 and AKT. Furthermore, a central role of L-type VGCC was demonstrated by the observation that siRNA to AKAP150 inhibited calcium oscillations. PKC is known to be activated by calcium, which can then activate MAPK and the PI3K/Akt [84]. PKC stimulates the ERK/MAPK and AKT pathway by phosphorylating and activating Raf-1 and AKT [85]. We showed that E_2_ and the ERβ agonists stimulated the phosphorylation of PKC, Raf-1 and AKT. Based on these observation, it is likely that the activation of the L-type VGCC by ERβ agonists leads to an increased calcium influx causing the phosphorylation and activation of PKC. The activated PKC can then phosphorylate Raf-1 and AKT activating the MAPK and AKT pathways. The activation of the MAPK and AKT can phosphorylate downstream factors, such as CREB, which was phosphorylated by the ERβ agonists. Our data is consistent with the observation that ERβ mediates the rapid phosphorylation of CREB in OVX mice [86].

ERβ is expressed in multiple regions in the brain [87], [88], [89], suggesting that it is involved in regulating neuronal processes. Our study demonstrates that ERβ activates different intracellular signaling proteins in neurons derived from ES cells, and that these pathways might be important regulators of neuronal activity in response to estrogens. The neurons derived from ES cells were glutamatergic and GABAergic, which we showed are a sensitive estrogen responsive neuronal-based system. While our studies demonstrate that ERβ activates nongenomic pathways in these cell types they cannot be generalized to other neuronal types, particularly those involved in hot flash prevention. In future studies, it should be possible to differentiate the ES to other neuronal subtypes, such as dopamine, norepinephrine and serotonin neurons to study the effects of the ERβ agonists. It will also be important to determine if a concentration-dependence exists over the physiological range of E_2_ and to understand the biological significance of ERβ-induced calcium regulation of oscillations and synchronizations in neurons derived from ES cells. One possibility is that the changes in calcium fluxes by ERβ agonists might regulate neurotransmitter secretion. Our findings indicate that neurons derived from ES cells might be useful to discover estrogens that directly regulate neuronal activity, and to investigate the differential roles of ERα and ERβ and mechanisms of actions of estrogens in neurons.

## Supporting Information

Figure S1 Synchronization analysis by random sample permutations. Histograms show the sum of [Ca2+]i peaks across 40–42 cells as a function of recording time. The orange line denotes the cutoff of random synchronization during the control period, and the red line denotes the cutoff of random synchronization during treatment period. The maximal peaks (cutoff) involved in a possible random synchronization are calculated by shuffling all [Ca2+]i peaks of the corresponding period for 2,000 times (p<0.01) using a Matlab script as described in Materials and Methods. (A) An example of increased synchronization frequency after 10 nM E2 exposure. 7 synchronization events during control period versus 18 synchronization events during E2 treatment period are recognized. (B) An example of unchanged synchronization frequency after 1 µM PPT treatment. 13 synchronizations during control period versus 16 synchronizations during PPT treatment period are recognized.
(0.62 MB TIF)

Figure S2 Tracings of [Ca2+]i oscillations in neurons derived from H9 cells exposed to ERα and/or ERβ agonists. After 10 min of baseline recording, neurons derived from H9 cells were treated with E2 (10 nM), ERB-041 (1 µM), DPN (1 µM), MF101 (125 µg/ml) and PPT (1 µM) and recorded for another 10 min. Pulsar identified peaks are labeled with “*”.
(0.23 MB TIF)

Figure S3 Tracings of [Ca2+]i oscillations in neurons derived from H7 cells exposed to ERα and/or ERβ agonists. After 10 min of baseline recording, neurons derived from H7 cells were treated with E2 (10 nM), ERB-041 (1 µM), DPN (1 µM), MF101 (125 µg/ml) and PPT (1 µM) and recorded for another 10 min. Pulsar identified peaks are labeled with “*”.
(0.24 MB TIF)

Figure S4 ERβ-selective ligands stimulated [Ca2+]i oscillations in neurons derived from the H7 cell line. Changes in the frequency of [Ca2+]i oscillations (A) and amplitudes of [Ca2+]i peaks (B) after treatment with E2 (10 nM), ERB-041 (1 µM), DPN (1 µM), MF101 (125 µg/ml), PPT (1 µM), PPT plus Ad-ERα (PPT+ERα) and control. The data after treatment (gray bars) are expressed relative to those before treatment (black bars), which are normalized to 1. The number of cells analyzed for the above groups were 200, 152, 174, 156, 202, 197 and 153, respectively from 3 independent experiments. **p<0.01, H7 neurons treated by different drugs are compared to control.
(0.16 MB TIF)

Figure S5 Immunofluorescent staining of neurons from mES cells with neuronal markers. The homogeneity of culture was shown by double staining of the cells with DAPI (blue) and Map2 (green), TujIII (green) or NeuN (red). The cells were stained for neuronal cytoskeleton markers, including Map2 (green), TujIII (red) and Tau (red) at day 5. Synaptic network was shown by white light (WL) image and Syp (red) clusters along neuronal processes. The cells developed voltage-gated channels, such as CaV1.2 (green). The cells displayed both GABAergic and glutamatergic properties as shown by immunofluorescence with Gad67 (red), GABA (green) and VGlut (red). Scale bar: 200 µm for Map2 and TujIII, 100 µm for NeuN in row 1, and 63 µm for the rest.
(6.37 MB TIF)

Figure S6 Neuronal excitability of neurons from mES cells with ion channel regulators. (A) Effects of KCl (56 mM), VTD (50 µM) and TTX (1 µM) on [Ca2+]i recorded from mES-neurons. “*” indicates Pulsar recognized [Ca2+]i peaks. (B–D): Changes in the frequency of [Ca2+]i oscillations (B) and amplitudes of [Ca2+]i peaks (C) after the above treatments (gray bars). The data were normalized to those at basal levels (black bars, assigned to 1). The number of cells for KCl, VTD, TTX and Control were 164, 156, 196 and 111, respectively from 2–4 independent experiments. ** p<0.01, neurons treated with drugs are compared to Control.
(0.22 MB TIF)

Figure S7 E2-induced increase of [Ca2+]i oscillations depends on extracellular Ca2+ source. (A) A representative example of a neuron from mES cells, which lost response to E2 in absence of extracellular Ca2+, and resumed the response after adding 2 mM Ca2+ back to the buffer. “*” indicates Pulsar recognized [Ca2+]i peaks. Changes in the frequency of [Ca2+]i oscillations (B), percentage of responsive cells (C) and amplitudes of [Ca2+]i peaks (D) after 10 nM E2 when deprived of Ca2+ (E2-Ca2+) or in presence of Ca2+ (E2+Ca2+). The data shown are the average of 140 cells from 3 independent experiments. ** p<0.01 when compared to basal levels.
(0.19 MB TIF)

Figure S8 Nifedipine abolishes E2-induced [Ca2+]i oscillations in neurons from mES cells. Upper tracing: after 10 min of control recordings, the cells were exposed to the L-type Ca2+ channel inhibitor, nifedipine (Nif, 10 µM) and recorded for another 10 min. Lower tracing: after 10 min of baseline recording with Nif (10 µM) treatment, the cells were exposed to 10 nM E2 in continuous presence of Nif and recorded for another 10 min. “*” indicates Pulsar recognized [Ca2+]i peaks.
(0.09 MB TIF)

Figure S9 Tracings of [Ca2+]i oscillations in neurons derived from mES cells in response to ERα and/or ERβ agonists. After 10 min of control recordings, neurons from mES cells were exposed to E2 (10 nM), ERB-041 (1 µM), DPN (1 µM), MF101 (125 µg/ml) and PPT (1 µM) and recorded for another 10 min. “*” indicates Pulsar recognized [Ca2+]i peaks.
(0.26 MB TIF)

Figure S10 ERβ-selective ligands stimulate [Ca2+]i oscillations in neurons from mES cells. Changes in the frequency of [Ca2+]i oscillations (A) and amplitudes of [Ca2+]i peaks (B) after treatment with E2 (10 nM), ERB-041 (1 µM), DPN (1 µM), MF101 (125 µg/ml), PPT (1 µM), PPT (10 µM), PPT plus overexpressed ERα (PPT + ERα), E2 plus overexpressed ERβ (E2 + ERβ) and non-treatment control. The data after treatment (gray bars) are expressed relative to cells before treatment (black bars), which are normalized to 1. The number of cells analyzed for the above groups were 307, 331, 337, 330, 296, 270, 288, 313 and 110, respectively from 8, 8, 6, 6, 6, 5, 6, 6 and 2 independent experiments. ** p<0.05, neurons treated by different drugs are compared to control.
(0.17 MB TIF)

Figure S11 Overexpression of ERα and ERβ in stable mES cells and their derived neurons. (A) Immunocytochemical staining with anti-flag of stable mES cells transfected with a plasmid expressing a flag-tagged ERα (ES-ERα) and their derived neurons (Neuron-ERα). Scale bar: 63 µM for ES-ERα, and 100 µM for Neuron-ERα. (B) Immunoprecipitation and western blot analysis of ERα expression (denoted by arrows) with anti-flag (left panel) and anti-ERα (right panel) in Neuron-ERα cells. Immunoprecipitation with IgG using the same amount of cells was used as negative control for antibody specificity. (C) Immunocytochemical staining with anti-flag of stable mES cells transfected with a plasmid expressing a flag-tagged ERβ (ES-ERβ) and their derived neurons (Neuron-ERβ). Scale bar: 63 µM for ES-ERβ, and 100 µM for Neuron-ERβ. (D) Immunoprecipitation and western blot analysis of ERβ expression (pointed by an arrow) with two antibodies against ERβ (sc-6821, Ab17) and anti-flag in NEU-ERβ. Immunoprecipitation with IgG from equal amount of cell lysates was used as negative control.
(1.99 MB TIF)

Figure S12 GT1-1 neurons infected with ERβ, but not ERα adenovirus respond to E2 treatment. (A) RNA levels of ERα and ERβ in GT1-1 cells at 48 h post-infection with adenovirus delivered ER (Ad-ERα or Ad-ERβ) at 5 and 10 MOI. The data shown are normalized to ER levels in GT1-1 cells infected with LacZ control viruses (Ad-LacZ, assigned to 1), and are the average of 3 independent experiments. Changes in the frequency of [Ca2+]i oscillations (B) and amplitudes of [Ca2+]i peaks (C) after E2 (10 nM) treatment in GT1-1 cells expressing ERα, ERβ or LacZ. The data after treatment (gray bars) are expressed relative to basal levels (black bars, normalized to 1). The numbers of cells analyzed for the above groups are 174, 164, 149, respectively from 4 or 5 independent experiments. ** p<0.01, Ad-ERα and Ad-ERβ cells compared to Ad-LacZ cells after E2 treatment.
(0.13 MB TIF)

Table S1 Sample size and p-value of calcium oscillations in figures.
(0.10 MB DOC)

Table S2 Sample size and p-value of calcium oscillations in supplementary figures.
(0.10 MB DOC)

Table S3 Sample size and p-value of signaling assays.
(0.08 MB DOC)
