# Supplementary figures and images for: Estrogen Receptor β-Selective Agonists Stimulate Calcium Oscillations in Human and Mouse Embryonic Stem Cell-Derived Neurons
Source: PLoS One. 2010 Jul 27;5(7):e11791. doi: 10.1371/journal.pone.0011791 (PMC2910705; doi:10.1371/journal.pone.0011791)

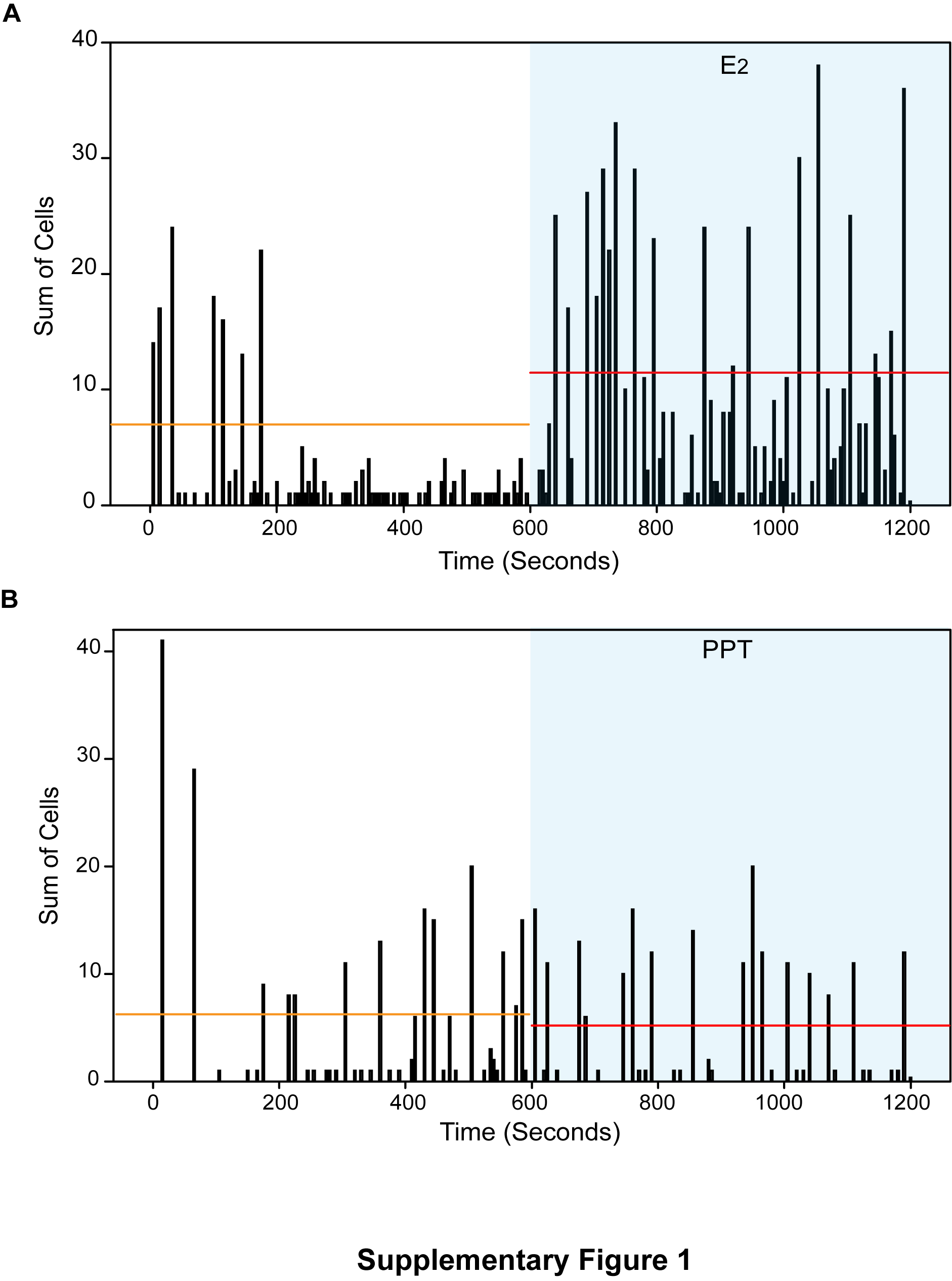

Supplement: Figure S1 — Synchronization analysis by random sample permutations. Histograms show the sum of [Ca2+]i peaks across 40–42 cells as a function of recording time. The orange line denotes the cutoff of random synchronization during the control period, and the red line denotes the cutoff of random synchronization during treatment period. The maximal peaks (cutoff) involved in a possible random synchronization are calculated by shuffling all [Ca2+]i peaks of the corresponding period for 2,000 times (p<0.01) using a Matlab script as described in Materials and Methods. (A) An example of increased synchronization frequency after 10 nM E2 exposure. 7 synchronization events during control period versus 18 synchronization events during E2 treatment period are recognized. (B) An example of unchanged synchronization frequency after 1 µM PPT treatment. 13 synchronizations during control period versus 16 synchronizations during PPT treatment period are recognized. (0.62 MB TIF) [file pone.0011791.s001.tif]

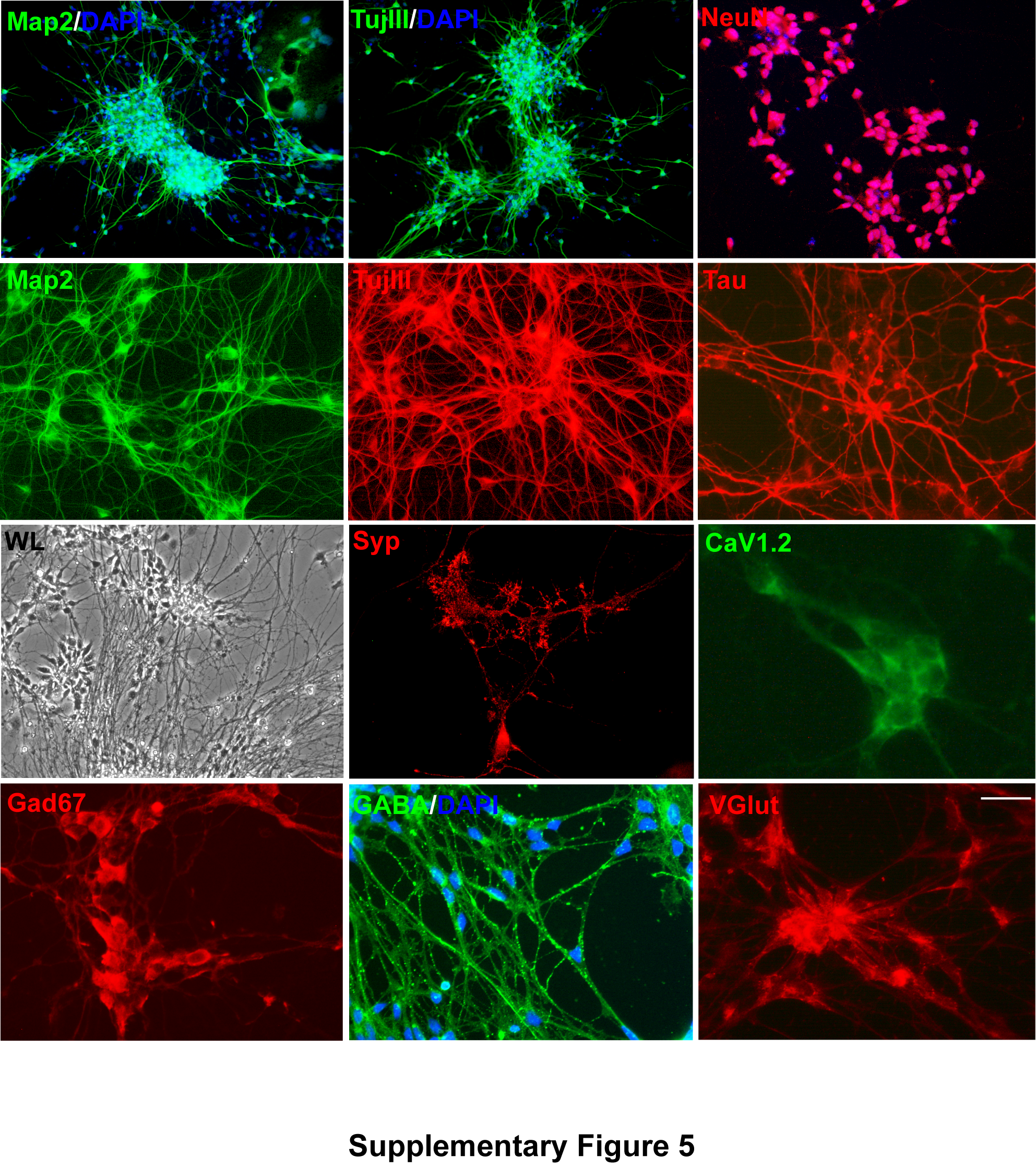

Supplement: Figure S5 — Immunofluorescent staining of neurons from mES cells with neuronal markers. The homogeneity of culture was shown by double staining of the cells with DAPI (blue) and Map2 (green), TujIII (green) or NeuN (red). The cells were stained for neuronal cytoskeleton markers, including Map2 (green), TujIII (red) and Tau (red) at day 5. Synaptic network was shown by white light (WL) image and Syp (red) clusters along neuronal processes. The cells developed voltage-gated channels, such as CaV1.2 (green). The cells displayed both GABAergic and glutamatergic properties as shown by immunofluorescence with Gad67 (red), GABA (green) and VGlut (red). Scale bar: 200 µm for Map2 and TujIII, 100 µm for NeuN in row 1, and 63 µm for the rest. (6.37 MB TIF) [file pone.0011791.s005.tif]

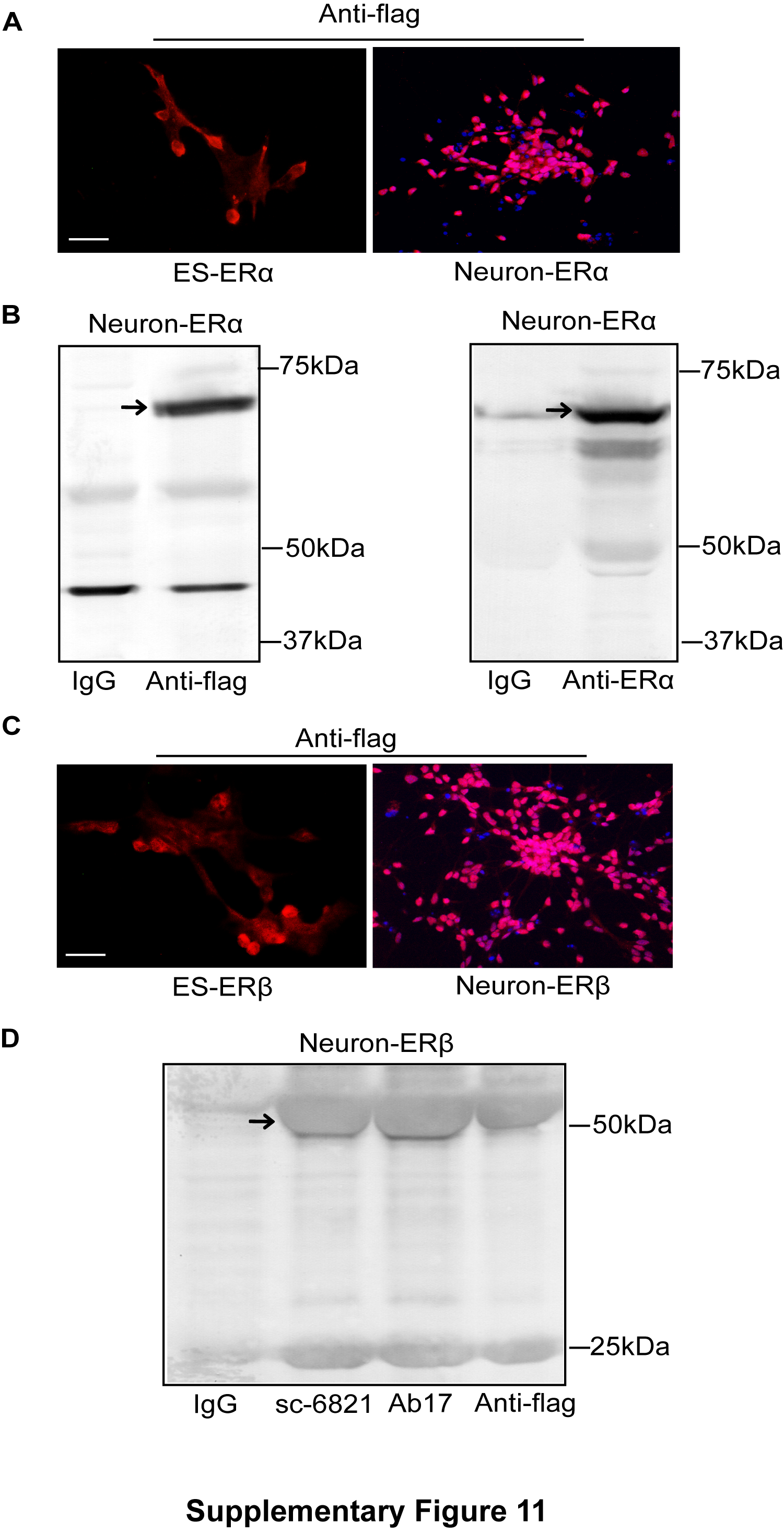

Supplement: Figure S11 — Overexpression of ERα and ERβ in stable mES cells and their derived neurons. (A) Immunocytochemical staining with anti-flag of stable mES cells transfected with a plasmid expressing a flag-tagged ERα (ES-ERα) and their derived neurons (Neuron-ERα). Scale bar: 63 µM for ES-ERα, and 100 µM for Neuron-ERα. (B) Immunoprecipitation and western blot analysis of ERα expression (denoted by arrows) with anti-flag (left panel) and anti-ERα (right panel) in Neuron-ERα cells. Immunoprecipitation with IgG using the same amount of cells was used as negative control for antibody specificity. (C) Immunocytochemical staining with anti-flag of stable mES cells transfected with a plasmid expressing a flag-tagged ERβ (ES-ERβ) and their derived neurons (Neuron-ERβ). Scale bar: 63 µM for ES-ERβ, and 100 µM for Neuron-ERβ. (D) Immunoprecipitation and western blot analysis of ERβ expression (pointed by an arrow) with two antibodies against ERβ (sc-6821, Ab17) and anti-flag in NEU-ERβ. Immunoprecipitation with IgG from equal amount of cell lysates was used as negative control. (1.99 MB TIF) [file pone.0011791.s011.tif]
