# Supplementary material for: Estrogen Receptor β-Selective Agonists Stimulate Calcium Oscillations in Human and Mouse Embryonic Stem Cell-Derived Neurons
Source: PLoS One. 2010 Jul 27;5(7):e11791. doi: 10.1371/journal.pone.0011791 (PMC2910705; doi:10.1371/journal.pone.0011791)
Supplement: Table S1 — Sample size and p-value of calcium oscillations in figures. (0.10 MB DOC) [file pone.0011791.s013.doc]

**Supplementary Table 1**: Sample size and p-value of calcium oscillations in figures.

| Figures | Treatments | Experiments | Cells | F/ t -value | p-value |
| --- | --- | --- | --- | --- | --- |
| Fig.2B | Treatment vs. Control | n/a | n/a | 122.69 | 2.20e-16 |
|  | KCl | 2 | 180 | 8.95 | 1.00e-08** |
|  | VTD | 2 | 175 | 6.16 | 1.00e-08** |
|  | TTX | 2 | 104 | -8.24 | 1.00e-08** |
|  | Control | 2 | 98 | n/a | n/a |
| Fig.2C | Treatment vs. Control | n/a | n/a | 114.36 | 2.20e-16 |
|  | KCl | 2 | 180 | 8.63 | 0.001** |
|  | VTD | 2 | 175 | 11.35 | 0.001** |
|  | TTX | 2 | 104 | -3.23 | 0.004** |
|  | Control | 2 | 98 | n/a | n/a |
| Fig.3B | Treatment vs. Control | n/a | n/a | 112.20 | 2.20e-16 |
|  | KCl | 2 | 164 | 9.12 | 1.00e-07** |
|  | VTD | 2 | 189 | 10.50 | 1.00e-07** |
|  | TTX | 2 | 172 | -6.10 | 1.00e-07** |
|  | Control | 3 | 153 | n/a | n/a |
| Fig.3C | Treatment vs. Control | n/a | n/a | 257.81 | 2.20e-16 |
|  | KCl | 2 | 164 | 7.28 | 1.00e-06** |
|  | VTD | 2 | 189 | 20.26 | 1.00e-06** |
|  | TTX | 2 | 172 | -5.39 | 1.00e-06** |
|  | Control | 3 | 153 | n/a | n/a |
| Fig.5A | Treatment vs. Control | n/a | n/a | 130.56 | 2.20e-16 |
|  | E_2_ | 2 | 143 | 15.32 | 0.001** |
|  | ERB-041 | 3 | 221 | 6.79 | 0.001** |
|  | DPN | 3 | 260 | 5.19 | 0.001** |
|  | MF101 | 3 | 188 | 3.61 | 0.002** |
|  | PPT | 2 | 134 | 1.39 | 0.474 |
|  | PPT+ERα | 3 | 179 | -0.03 | 1 |
|  | Control | 2 | 98 | n/a | n/a |
| Fig. 5B | Treatment vs. Control | n/a | n/a | 72.21 | 2.20e-16 |
|  | E_2_ | 2 | 143 | 9.89 | 0.001** |
|  | ERB-041 | 3 | 221 | 8.69 | 0.001** |
|  | DPN | 3 | 260 | 6.46 | 0.001** |
|  | MF101 | 3 | 188 | 3.72 | 0.001** |
|  | PPT | 2 | 134 | 2.14 | 0.121 |
|  | PPT+ERα | 3 | 179 | 0.73 | 0.917 |
|  | Control | 2 | 98 | n/a | n/a |
| Fig.7A | Treatment vs. Control | n/a | n/a | 78.20 | 2.20e-16 |
|  | Nif | 5 | 233 | -2.32 | 0.100 |
|  | Nif+E_2_ | 4 | 213 | 0.88 | 0.900 |
|  | AgTx | 3 | 163 | -5.44 | 0.001** |
|  | AgTx+E_2_ | 3 | 170 | 11.58 | 0.001** |
|  | CgTx | 3 | 165 | -3.02 | 0.016* |
|  | CgTx+E_2_ | 3 | 156 | 3.82 | 0.001** |
|  | E_2_-BSA | 3 | 139 | 5.76 | 0.001** |
|  | E_2_ | 3 | 113 | 7.82 | 0.001** |
|  | Control | 2 | 99 | n/a | n/a |
| Fig.7B | Treatment vs. Control | n/a | n/a | 24.68 | 2.20e-16 |
|  | Nif | 5 | 233 | 4.29 | 0.001** |
|  | Nif+E_2_ | 4 | 213 | 5.15 | 0.001** |
|  | AgTx | 3 | 163 | 2.32 | 0.100 |
|  | AgTx+E_2_ | 3 | 170 | 7.26 | 0.001** |
|  | CgTx | 3 | 165 | 3.66 | 0.002** |
|  | CgTx+E_2_ | 3 | 156 | 4.95 | 0.001** |
|  | E_2_-BSA | 3 | 139 | 7.22 | 0.001** |
|  | E_2_ | 3 | 113 | 12.18 | 0.001** |
|  | Control | 2 | 99 | n/a | n/a |
| Fig.8C | Treatment vs. Control | n/a | n/a | 165.53 | 2.20e-16 |
|  | Ad-si-AKAP | 7 | 366 | 4.65 | 1.03e-05** |
|  | Ad-si-Luc | 6 | 286 | 15.63 | 1.00e-05** |
|  | Control | 6 | 320 | n/a | n/a |
|  | Ad-si-AKAP vs. Ad-si-Luc | n/a | n/a | -11.60 | 1.00e-05** |
| Fig.8D | Treatment vs. Control | n/a | n/a | 154.51 | 2.20e-16 |
|  | Ad-si-AKAP | 7 | 366 | 8.68 | 1.00e-10** |
|  | Ad-si-Luc | 6 | 286 | 15.68 | 1.00e-10** |
|  | Control | 6 | 320 | n/a | n/a |
|  | Ad-si-AKAP vs. Ad-si-Luc | n/a | n/a | -7.75 | 1.00e-10** |

** p < 0.01, * p < 0.05 as labeled in Figures.
