# Supplementary material for: Estrogen Receptor β-Selective Agonists Stimulate Calcium Oscillations in Human and Mouse Embryonic Stem Cell-Derived Neurons
Source: PLoS One. 2010 Jul 27;5(7):e11791. doi: 10.1371/journal.pone.0011791 (PMC2910705; doi:10.1371/journal.pone.0011791)
Supplement: Table S2 — Sample size and p-value of calcium oscillations in supplementary figures. (0.10 MB DOC) [file pone.0011791.s014.doc]

**Supplementary Table 2**: Sample size and p-value of calcium oscillations in supplementary figures.

| Figures | Treatments | Experiments | Cells | F/ t -value | p-value |
| --- | --- | --- | --- | --- | --- |
| Fig.S4A | Treatment vs. Control | n/a | n/a | 47.57 | 2.20e-16 |
|  | E_2_ | 3 | 200 | 8.92 | 1.00e-04** |
|  | ERB-041 | 3 | 152 | 4.73 | 1.00e-04** |
|  | DPN | 3 | 174 | 7.78 | 1.00e-04** |
|  | MF101 | 3 | 156 | 6.14 | 1.00e-04** |
|  | PPT | 3 | 202 | 0.31 | 0.999 |
|  | PPT+ERα | 3 | 197 | 1.12 | 0.722 |
|  | Control | 3 | 153 | n/a | n/a |
| Fig.S4B | Treatment vs. Control | n/a | n/a | 66.34 | 2.20e-16 |
|  | E_2_ | 3 | 200 | 7.50 | 0.001** |
|  | ERB-041 | 3 | 152 | 10.73 | 0.001** |
|  | DPN | 3 | 174 | 7.44 | 0.001** |
|  | MF101 | 3 | 156 | 6.00 | 0.001** |
|  | PPT | 3 | 202 | 2.34 | 0.087 |
|  | PPT+ERα | 3 | 197 | 3.30 | 0.005** |
|  | Control | 3 | 153 | n/a | n/a |
| Fig.S6B | Treatment vs. Control | n/a | n/a | 221.41 | 2.20e-16 |
|  | KCl | 3 | 164 | 7.46 | 1.00e-10** |
|  | VTD | 3 | 156 | 8.10 | 1.00e-10** |
|  | TTX | 4 | 196 | -14.49 | 1.00e-10** |
|  | Control | 2 | 111 | n/a | n/a |
| Fig.S6C | Treatment vs. Control | n/a | n/a | 243.76 | 2.20e-16 |
|  | KCl | 3 | 164 | 7.40 | 1.00e-10** |
|  | VTD | 3 | 156 | 7.82 | 1.00e-10** |
|  | TTX | 4 | 196 | -15.63 | 1.00e-10** |
|  | Control | 2 | 111 | n/a | n/a |
| Fig.S7B | Treatment vs. Control | 3 | 140 | 249.65 | 2.20e-16** |
| Fig.S7C | Treatment vs. Control | n/a | n/a | 415.03 | 2.30e-05 |
|  | E_2_+Ca^2+^ vs. E_2_-Ca^2+^ | 3 | 140 | 17.75 | 5.92e-05** |
| Fig.S7D | Treatment vs. Control | 3 | 140 | 205.66 | 2.20e-16** |
| Fig.S10A | Treatment vs. Control | n/a | n/a | 172.27 | 2.20e-16 |
|  | E_2_ | 8 | 307 | 8.68 | 0.001** |
|  | ERB-041 | 8 | 331 | 14.00 | 0.001** |
|  | DPN | 6 | 337 | 9.60 | 0.001** |
|  | MF101 | 6 | 330 | 9.24 | 0.001** |
|  | PPT | 6 | 296 | 0.95 | 0.791 |
|  | PPT_10uM_ | 5 | 270 | 1.41 | 0.456 |
|  | PPT+ERα | 6 | 288 | 1.89 | 0.207 |
|  | E_2_+ERβ | 6 | 313 | 14.00 | 0.001** |
|  | Control | 2 | 110 | n/a | n/a |
| Fig.S10B | Treatment vs. Control | n/a | n/a | 124.45 | 2.20e-16 |
|  | E_2_ | 8 | 307 | 9.68 | 0.001** |
|  | ERB-041 | 8 | 331 | 10.07 | 0.001** |
|  | DPN | 6 | 337 | 6.16 | 0.001** |
|  | MF101 | 6 | 330 | 6.94 | 0.001** |
|  | PPT | 6 | 296 | 3.04 | 0.012* |
|  | PPT_10uM_ | 5 | 270 | 1.26 | 0.563 |
|  | PPT+ERα | 6 | 288 | 1.02 | 0.742 |
|  | E_2_+ERβ | 6 | 313 | 11.88 | 0.001** |
|  | Control | 2 | 110 | n/a | n/a |
| Fig. S12B | Treatment vs. Ad-LacZ | n/a | n/a | 79.15 | 2.20e-16 |
|  | Ad-ERα | 5 | 174 | -1.75 | 0.141 |
|  | Ad-ERβ | 5 | 164 | 10.99 | 1.00e-10** |
|  | Ad-LacZ | 4 | 149 | n/a | n/a |
| Fig.S12C | Treatment vs. Ad-LacZ | n/a | n/a | 75.58 | 2.20e-16 |
|  | Ad-ERα | 5 | 174 | -1.49 | 0.233 |
|  | Ad-ERβ | 5 | 164 | 9.49 | 1.00e-10** |
|  | Ad-LacZ | 4 | 149 | n/a | n/a |

** p < 0.01, * p < 0.05 as labeled in Figures.
