# Supplementary material for: Estrogen Receptor β-Selective Agonists Stimulate Calcium Oscillations in Human and Mouse Embryonic Stem Cell-Derived Neurons
Source: PLoS One. 2010 Jul 27;5(7):e11791. doi: 10.1371/journal.pone.0011791 (PMC2910705; doi:10.1371/journal.pone.0011791)
Supplement: Table S3 — Sample size and p-value of signaling assays. (0.08 MB DOC) [file pone.0011791.s015.doc]

**Supplementary Table 3**: Sample size and p-value of signaling assays.

| Figures | Treatments | Experiments | F/t-value | p-value |
| --- | --- | --- | --- | --- |
| Fig.9A | Treatment vs. 0 min | n/a | 203.01 | 7.42e-10 |
| PKC-P | 5 min | 2 | 0.73 | 0.921 |
|  | 10 min | 3 | 4.00 | 0.010** |
|  | 15 min | 3 | 5.35 | 0.001** |
|  | 20 min | 3 | 4.12 | 0.008** |
|  | 30 min | 2 | 3.41 | 0.026* |
| Fig.9B | Treatment vs. 0 min | n/a | 145.68 | 2.49e-08 |
| AKT-P | 10 min | 3 | 3.32 | 0.029* |
|  | 20 min | 3 | 3.75 | 0.015* |
|  | 30 min | 3 | 4.77 | 0.004** |
|  | 45 min | 2 | 2.18 | 0.166 |
| Fig.9C | Treatment vs. 0 min | n/a | 362.46 | 1.76e-13 |
| RAF-P | 5 min | 2 | 2.60 | 0.070 |
|  | 10 min | 4 | 5.78 | 0.001** |
|  | 20 min | 4 | 7.55 | 0.001** |
|  | 30 min | 4 | 6.12 | 0.001** |
| Fig.9D | Treatment vs. 0 min | n/a | 87.13 | 6.48e-08 |
| ERK1-P | 10 min | 3 | 2.36 | 0.118 |
|  | 20 min | 3 | 2.81 | 0.057 |
|  | 30 min | 3 | 4.58 | 0.004** |
|  | 45 min | 3 | 3.24 | 0.028* |
| Fig.9D | Treatment vs. 0 min | n/a | 141.22 | 6.12e-09 |
| ERK2-P | 10 min | 3 | 2.95 | 0.045* |
|  | 20 min | 3 | 3.97 | 0.009** |
|  | 30 min | 3 | 6.11 | 0.001** |
|  | 45 min | 3 | 4.00 | 0.008** |
| Fig.9E | Treatment vs. 0 min | n/a | 66.55 | 3.62e-09 |
| CREB-P | 15 min | 3 | 2.09 | 0.217 |
|  | 30 min | 3 | 4.34 | 0.004** |
|  | 45 min | 3 | 4.09 | 0.006** |
|  | 60 min | 3 | 3.56 | 0.016* |
|  | 90 min | 3 | 2.55 | 0.101 |
|  | 120 min | 2 | 1.58 | 0.459 |
| Fig.10A | Treatment vs. Control | n/a | 895.04 | 2.20e-16 |
| AKT-P | E_2_ | 4 | 13.15 | 1.00e-04** |
|  | ERB-041 | 4 | 12.79 | 1.00e-04** |
|  | DPN | 4 | 7.63 | 1.00e-04** |
|  | MF101 | 3 | 4.89 | 0.001** |
|  | PPT | 3 | 0.72 | 0.926 |
| Fig.10B | Treatment vs. Control | n/a | 45.84 | 6.12e-10 |
| ERK1-P | E_2_ | 4 | 3.28 | 0.017** |
|  | ERB-041 | 4 | 3.87 | 0.005** |
|  | DPN | 4 | 2.22 | 0.141 |
|  | MF101 | 4 | 2.12 | 0.168 |
|  | PPT | 4 | 0.47 | 0.986 |
| Fig.10B | Treatment vs. Control | n/a | 79.71 | 5.60e-12 |
| ERK2-P | E_2_ | 4 | 4.44 | 0.001** |
|  | ERB-041 | 4 | 5.15 | 0.001** |
|  | DPN | 4 | 2.85 | 0.042* |
|  | MF101 | 4 | 2.35 | 0.111 |
|  | PPT | 4 | 0.05 | 1.000 |
| Fig.10C | Treatment vs. Control | n/a | 305.43 | 2.20e-16 |
| CREB-P | E_2_ | 4 | 6.34 | 0.001** |
|  | ERB-041 | 4 | 8.44 | 0.001** |
|  | DPN | 4 | 5.11 | 0.001** |
|  | MF101 | 4 | 3.73 | 0.004** |
|  | PPT | 4 | 0.50 | 0.981 |

** p < 0.01, * p < 0.05 as labeled in Figures.
